# Increased dietary protein stimulates amino acid catabolism via the gut microbiota and secondary bile acid production

**DOI:** 10.1080/19490976.2025.2465896

**Published:** 2025-02-20

**Authors:** Sandra Tobón-Cornejo, Monica Sanchez-Tapia, Rocio Guizar-Heredia, Laura Velázquez Villegas, Lilia G. Noriega, Janette Furuzawa-Carballeda, Rogelio Hernández-Pando, Natalia Vázquez-Manjarrez, Omar Granados-Portillo, Adriana López-Barradas, Rosa Rebollar-Vega, Otoniel Maya, Aaron W. Miller, Aurora Serralde, Martha Guevara-Cruz, Nimbe Torres, Armando R. Tovar

**Affiliations:** a Departamento de Fisiología de la Nutrición, Instituto Nacional de Ciencias Médicas y Nutrición Salvador Zubirán, México City, México; b Departamento de Cirugía Experimental, Instituto Nacional de Ciencias Médicas y Nutrición Salvador Zubirán, México City, México; c Departamento de Patología Experimental, Instituto Nacional de Ciencias Médicas y Nutrición Salvador Zubirán, México City, México; d RED de apoyo a la investigación, Coordinación de la Investrigación Científica, UNAM e Instituto Nacional de Ciencias Médicas y Nutrición Salvador Zubirán, México City, México; e Physics Department, Chalmers University of Technology, Chalmers E-Commons, Gothenburg, Sweden; f Department of Inflammation and Immunity, Lerner Research Institute, Cleveland Clinic, Cleveland, OH, USA; g Departamento de Nutrición Clínica, Instituto Nacional de Ciencias Médicas y Nutrición Salvador Zubirán, México City, México

**Keywords:** Gut microbiota, high-protein diet, secondary bile acids, amino acid catabolism, glucagon

## Abstract

Excess amino acids from a protein-rich diet are mainly catabolized in the liver. However, it is still unclear to what extent the gut microbiota may be involved in the mechanisms governing this catabolism. Therefore, the aim of this study was to investigate whether consumption of different dietary protein concentrations induces changes in the taxonomy of the gut microbiota, which may contribute to the regulation of hepatic amino acid catabolism. Consumption of a high-protein diet caused overexpression of HIF-1α in the colon and increase in mitochondrial activity, creating a more anaerobic environment that was associated with changes in the taxonomy of the gut microbiota promoting an increase in the synthesis of secondary bile acids, increased secretion of pancreatic glucagon. This effect was demonstrated in pancreatic islets, where secondary bile acids stimulated the expression of the PC2 enzyme that promotes glucagon formation. The increase in circulating glucagon was associated with an induction of the expression of hepatic amino acid-degrading enzymes, an effect attenuated by antibiotics. Thus, high protein intake in mice and humans induced the increase of different species in the gut microbiota with the capacity to produce secondary bile acids leading to an increase in secondary bile acids and glucagon levels, promoting amino acid catabolism.

## GRAPHICAL ABSTRACT

**Figure:**
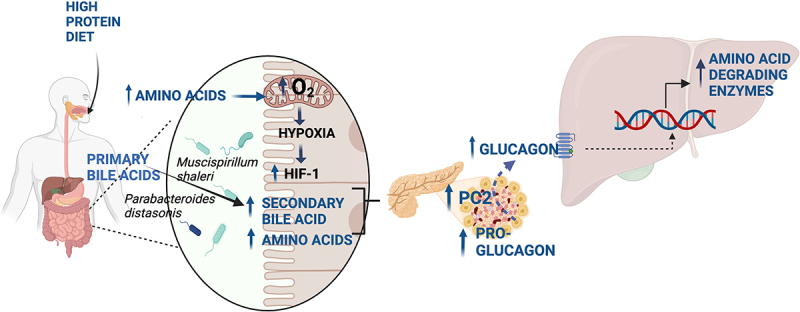

## 1. Introduction

Dietary proteins are macronutrients that play an essential role in human nutrition.^1^ The type and concentration of protein could be modified as part of different dietary strategies for the treatment of various pathologies to produce health benefits.^2^ In everyday life, there is an inadequate consumption of protein according to recommendations in different population groups. For many decades, efforts have been made to study the consequences of consuming low, adequate or high protein diets in animal models.^3–5^ Dietary protein provides the organism with amino acids to maintain protein synthesis in all cells and to become precursors of several molecules with diverse biological functions. However, when protein intake exceeds requirements, the fate of amino acids is to be oxidized, channeled to gluconeogenesis and the tricarboxylic acid cycle to generate ATP.^6^ In addition, the consumption of a high protein diet is accompanied by an increased excretion of nitrogen as urea.^7^

Among the adaptations that have been demonstrated due to different dietary protein concentration-dependent changes are variations in several circulating hormones such as insulin, glucagon, and glucocorticoids.^8,9^ In particular, an increase in glucagon is observed in high protein diets, which is associated with an increase in the transcription of amino acid degrading enzymes (AADE) genes, mainly through the transcription factor cAMP response element binding protein (CREBP),^10^ which accelerates the catabolism of the excess amino acids.^11^ Another factor that also stimulates the transcription of these enzymes is hepatocyte nuclear factor 4 alpha (HNF4α).^12,13^ Under these conditions, the expression of some urea cycle enzyme genes is also induced to eliminate excess amino acids.^12^

Recently, it has been recognized that other factors are involved in the adaptation to the consumption of different dietary protein concentrations,^2^ including adaptations that occur in the gastrointestinal tract, which may be associated with changes in the gut microbiota.^6,14^ Several studies in animal models have shown that consumption of low or high protein diets can alter the abundance of some species of the gut microbiota.^15,16^ It is known that the consumption of a high-protein diet can increase the concentration of intestinal amino acids and peptides produced by processing of intestinal enzymes that can reach the colon, and these are used as substrates by several bacteria, promoting their abundance.^14,17^ On the other hand, it has been shown that the amount of protein in the diet is associated with a decrease in carbohydrates, and this reduction may also affect the abundance of specific species of gut microbiota that are highly dependent on this metabolic substrate.^14^ As a consequence, several metabolites have been shown to increase after consumption of a high-protein diet, including phenols, indoles, amines, sulfides, and short-chain fatty acids, among others, which may have health implications for the host.^18–20^

Consumption of a high-protein diet is associated with an increase in circulating levels of amino acids.^2^ Elevation of some amino acids has been associated with various toxic effects,^21^ thus amino acid catabolism must be activated to reduce excess amino acids in serum and tissues.^22,23^ However, it has not been established whether there is an axis between the gut and the hepatic amino acid catabolism involving taxonomic adaptations of the gut microbiota as part of the mechanism of hepatic amino acid catabolism to reach a steady state of amino acid concentration after consumption of a protein-rich diet.

Therefore, the aim of the present study was to assess whether the consumption of a high-protein diet can modify the gut microbiota, leading to changes in some metabolites that may in turn trigger signals to promote amino acid catabolism in the liver, thereby facilitating a physiological adaptation of the host.

## 2. Results

### 2.1. Consumption of a high-protein diet reduced body fat and increased energy expenditure

**Figure 1.**
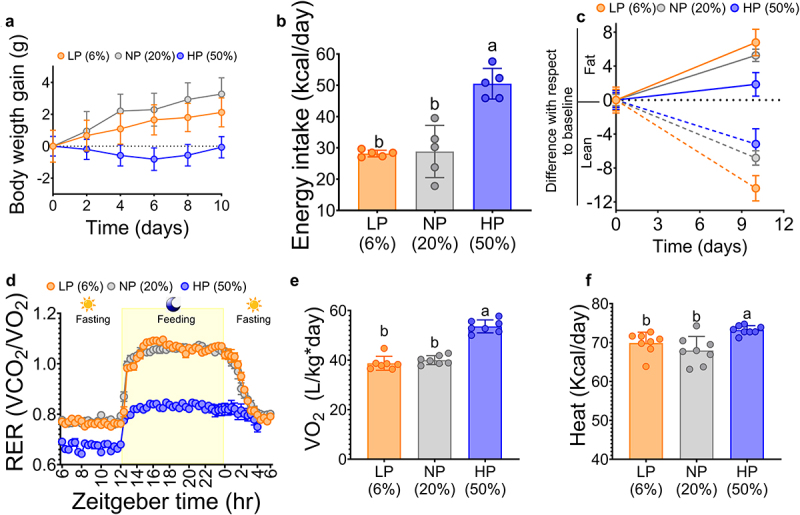
Mice fed increasing concentrations of dietary protein had decreased body fat mass and increased energy expenditure. (a) Body weight gain, (b) energy intake, (c) fat mass and lean mass, (d) respiratory exchange ratio (RER), (e) oxygen consumption, and (f) heat after dietary protein consumption. LP (low protein), NP (normal protein), HP (high protein). Data are expressed as mean ± SEM. Statistical analyses were performed by two-way ANOVA followed by Tukey’s post hoc test (for C and E-F). Multiple comparisons are shown in lowercase (a > b), n=5-8.

To evaluate metabolic adaptations to different dietary protein concentrations, mice were fed a low (6%), adequate (20%), or high (50%) protein diet for 10 days. The data showed that mice fed an adequate protein diet gained more body weight than the other two groups. However, it was evident that those fed the high-protein diet had a decrease in body weight gain, reaching almost the initial body weight after 10 days of dietary treatment (Figure 1a). This body weight behavior occurred despite the fact that mice fed the high-protein diet had the highest dietary protein intake and energy intake expressed in Kcal/day (Figure 1b). Analysis of body composition revealed that consumption of a high-protein diet was associated with the lowest increase in body fat mass, while maintaining lean body mass similar to that of mice fed an adequate protein diet (Figure 1c). Respiratory exchange ratio (RER) was measured to assess energy expenditure and the fuel metabolized to supply energy to the body. It was observed that mice fed 50% dietary protein had the lowest RER (0.7) compared to the other groups (0.8). However, during the feeding period, mice fed 6% or 20% protein diet reached an RER value of about 1. In contrast, mice fed 50% protein diet increased the RER value to about 0.8, suggesting that the main source of energy was dietary protein (Figure 1d). Interestingly, despite the low RER, mice fed 50% protein in the diet had the highest oxygen consumption during the 24-hour period compared to the other groups (Figure 1e). Similarly, mice fed 50% protein in the diet had the highest energy expenditure expressed as heat (Figure 1f).

### 2.2. Consumption of a high protein diet modified the transcriptome of the mouse colon

**Figure 2.**
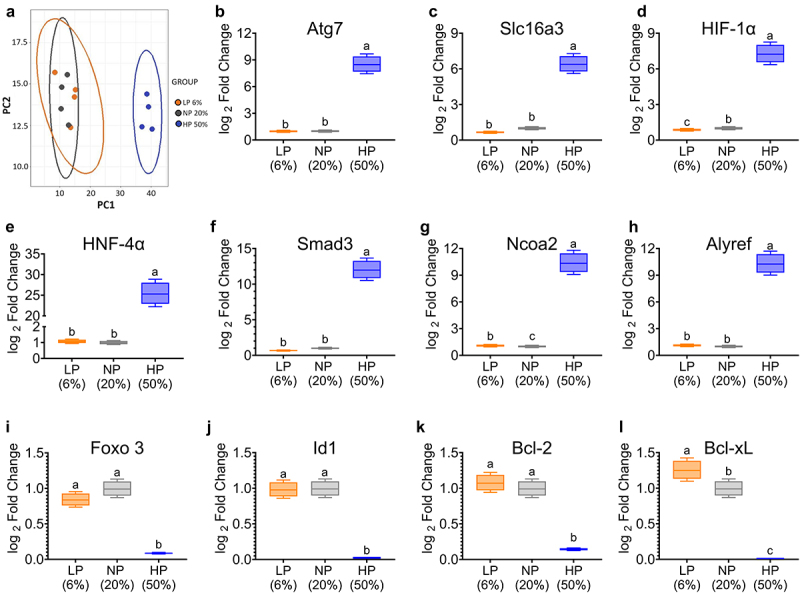
Differential gene expression changes in the colon after feeding different protein concentrations. (a) Principal component analysis (PCA) plot based on differential mRNA expression in mice fed different protein concentrations. Relative abundance of mRNAs with significant changes: (b) autophagy related 7 (*Atg7*), (c) monocarboxylate transporter 4 (MCT4 or *Slc16a3*), (d) hypoxia inducible factor-1 (*Hif-1α*), (e) hepatocyte nuclear factor 4a (*HNF-4A*), (f) Mothers against decapentaplegic homolog 3 (*Smad3*), (g) Nuclear receptor coactivator 2 (*Ncoa2*), (h) Aly/REF export factor (*Alyref*), (i) Forkhead box O3 (*Foxo 3*). (j) Inhibitor of differentiation 1 (*Id1*), (k) B-cell lymphoma 2 (*Bcl-2*), and (l) B-cell lymphoma extra large (*Bcl-xL*). LP (low protein), NP (normal protein), HP (high protein). Statistical analyses were performed by two-way ANOVA followed by Tukey’s post hoc test (B-L). Multiple comparisons are summarized with lowercase letters (a > b). n=5.

**Figure 3.**
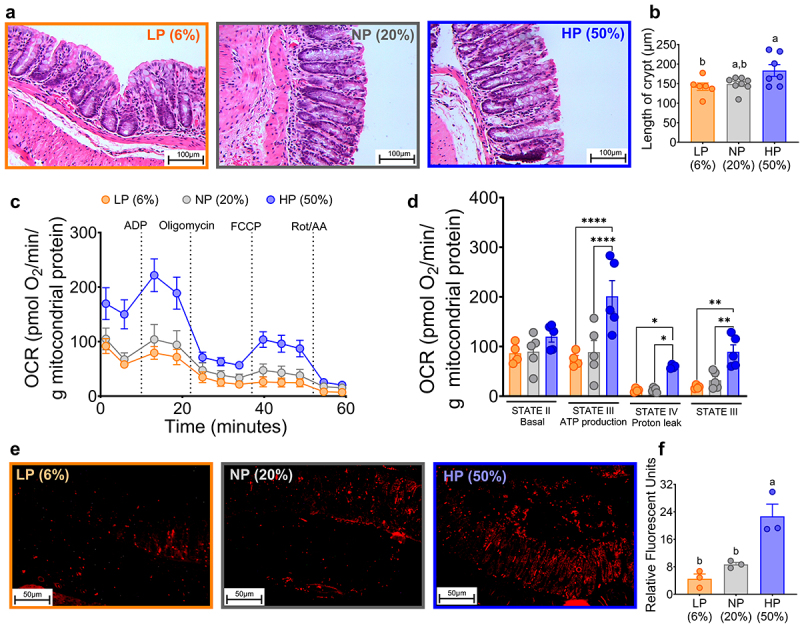
Colon morphology, mitochondrial function, and hypoxic state were altered in mice fed a high-protein diet. (a) Histologic sections of ascending colon from animals fed different protein concentrations. (b) Crypt length analyzed by automated morphometric analysis (hematoxylin-eosin stained micrographs, magnification ×100) (n=8-10). (c and d) Mitochondrial respiratory states of isolated colonic mitochondria. Values were obtained from oxygen respiratory rate (OCR), (n=4). (e) Colonic hypoxic microenvironment of mice fed different protein concentrations injected with pimonidazole. (f) Relative fluorescence units of colon, immunofluorescence staining was analyzed with imageJ (n=3) for hypoxia assessment. LP (low protein), NP (normal protein), HP (high protein). Data are expressed as mean ± SEM. Densitometric statistical analyses were performed by two-way ANOVA followed by Tukey’s post hoc test. Multiple comparisons are summarized with lowercase letters (a > b) (for B and F). p < 0.05; **p < 0.01; ***p < 0.001; ****p < 0.0001. n = 4 (for D).

First, we investigated whether the consumption of different concentrations of dietary protein could modulate the transcriptome in the ileum and colon. For this purpose, an RNA-seq assay was performed in both parts of the intestine. In particular, the transcriptome in the ileum was similar in all three groups. However, Principal Coordinate Analysis (PCoA) revealed that in the colon, those fed 50% dietary protein had a different gene expression profile than the 6% and 20% groups (Figure 2a). Volcano plot analysis showed that mice fed 50% dietary protein had several genes that were significantly over- or under-expressed. In particular, *Atg7*, *Slc16A3*, *HIF1α*, *HNF4α*, *Smad3*, *Ncoa2*, and *Alyref* were 9- to 25-fold overexpressed (Figure 2b–h). In contrast, *Foxo3*, *Id1*, *Bcl-2* and *Bcl-xL* were strongly under-expressed (Figure 2i–l). Some of these genes are associated with cell replication and metabolism. In fact, histological studies showed that the length of Lieberkühn crypts was similar between animals fed a low and adequate protein diet, while the group of animals fed a high protein diet had significantly longer crypts with a cellular constitution apparently similar to that of the other groups fed 6% or 20% dietary protein (Figure 3a,b), suggesting an accelerated process of cell replication, as some amino acids, particularly glutamine, have been shown to play a key role in enterocyte energy metabolism, which in turn accelerates protein turnover in these cells.^24^

### 2.3. Consumption of a high-protein diet produced an increased colonic hypoxia associated with an increase in mitochondrial function

Changes in gene expression of *HIF-1α* are indicative of oxygen utilization, particularly for mitochondrial respiration of isolated mitochondria obtained from the colon. Indeed, in mice fed 50% dietary protein, mitochondria isolated from the colon exhibited a significant increase in the rate of oxygen consumption associated with ATP production, as well as proton leak and non-mitochondrial respiration (Figure 3c,d). In fact, assessment of the anaerobic state in the colon measured with pimidazole, a probe to assess the degree of oxygenation in the colon, showed that consumption of 50% dietary protein produced a nearly 3-fold greater hypoxic state compared to those fed 6% or 20% dietary protein (Figure 3e,f).

### 2.4. The hypoxic state in the colon induced by a high-protein diet altered the gut microbiota

**Figure 4.**
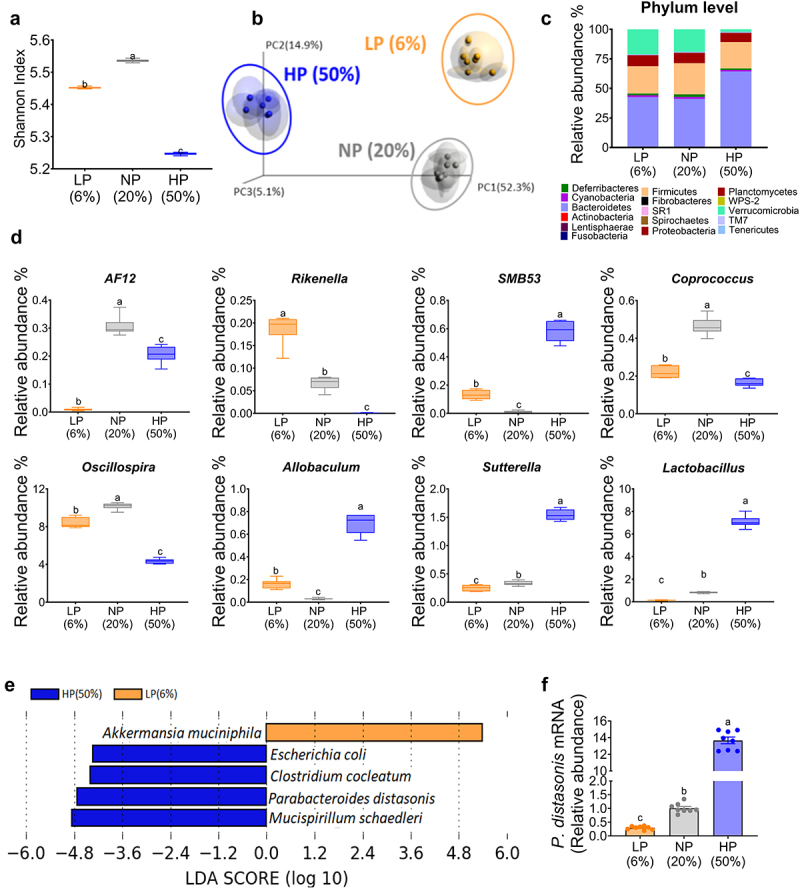
Gut microbiota is modified by dietary protein concentration in mice. (a) Comparative alpha diversity of gut microbiota, (b) Principal coordinate analysis of beta diversity. Taxonomy of gut microbiota at the level of (c) phylum and (d) genus; (e) linear discriminant analysis (LDA) shows significant differences in the relative abundance of bacterial species in mice fed different protein concentrations. (f) Relative mRNA abundance of *P. distasonis*. LP (low protein), NP (normal protein), HP (high protein). Statistical analyses were performed by two-way ANOVA followed by Tukey’s post hoc test (for A and D). Multiple comparisons are indicated by lowercase letters (a > b > c); n=8-10. Data are expressed as mean ± SEM.

Due to changes in the expression of some genes such as *HIF-1α* in the colon and an increase in the oxygen consumption rate (OCR) due to changes in mitochondrial activity, especially in those animals fed a high protein diet, this suggested that there may be changes in the aerobic/anaerobic state in the colon, which would possibly have repercussions on the taxonomy of the gut microbiota. To this end, we evaluated the gut microbiota by sequencing the 16S ribosomal RNA gene. The results showed that the diversity of the gut microbiota was modified according to the amount of protein consumed in the diet; in particular, animals fed a 50% protein diet showed a small decrease in the Shannon index, an indicator of gut microbiota diversity (Figure 4a). However, principal component analysis of the gut microbiota, which is an indicator of beta diversity, showed that the gut microbiota changed not only in quantitative abundance but also in the type of bacteria present in the colon depending on the amount of protein in the diet (Figure 4b). Taxonomic analysis showed that consumption of a diet with 50% dietary protein increased the Bacteroidetes phylum (Figure 4c), which affected the gut microbiota at the genus level (Figure 4d). Additionally, the LDA score analysis showed that the species that significantly increased with the consumption of a 50% protein diet were *Escherichia coli*, *Clostridium cocleatum*, *Mucispirillum schaedleri* and *Parabacteroides distasonis*, while at 6% protein it was *Akkermansia muciniphila* (Figure 4e). Particularly, the qPCR analysis showed that consumption of 50% dietary protein increased by 12. 6- and 46-fold compared to those consuming 20% or 6% dietary protein, respectively (Figure 4f).

### 2.5. Modulation of the gut microbiota by a high protein diet was associated with an increase in serum secondary bile acid and glucagon concentrations

**Figure 5.**
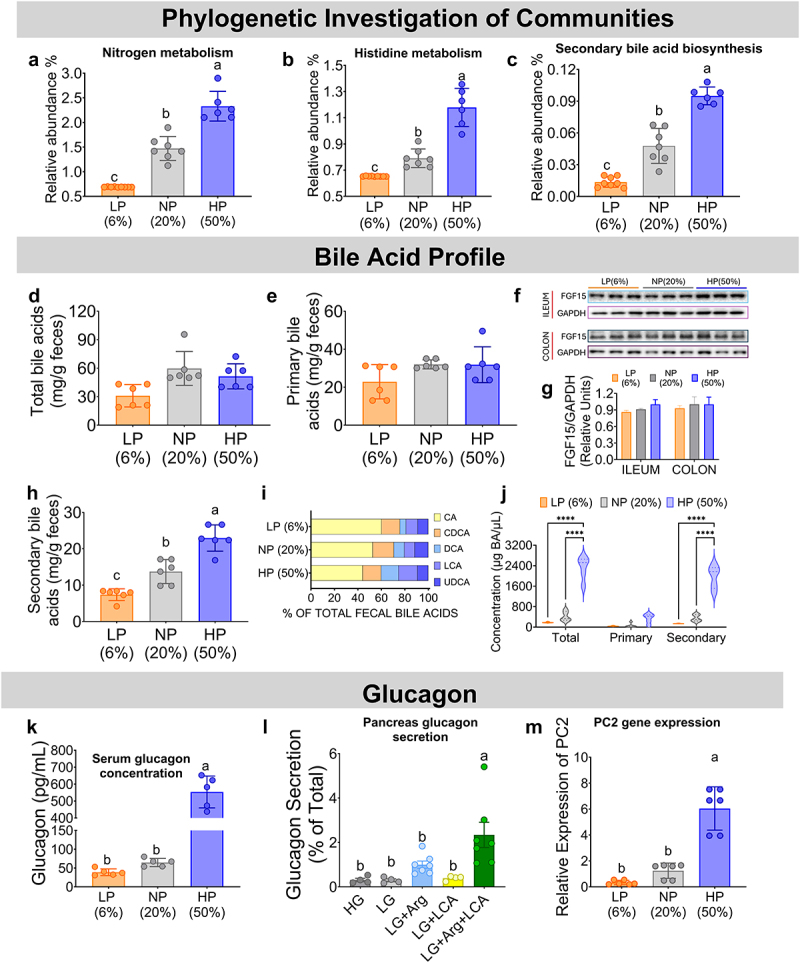
Impact of Dietary Protein Levels on Gut Microbiota Potential Functions in Mice. Phylogenetic analysis of communities to predict the functional metagenome and thus differentially active bacterial metabolic pathways, including (a) nitrogen metabolism, (b) histidine metabolism, and (c) secondary bile acid biosynthesis after consumption of different dietary protein concentrations. (d) Total fecal concentration of bile acids, (e) primary fecal bile acids concentration, (f) western blot and (g) densitometric analysis of FGF15 in ileum and colon, (H) fecal secondary bile acid concentrations, (I) bile acid profile by percentage. (j) serum bile acids concentration, (j) serum glucagon concentration, (l) stimulation of glucagon secretion, and (m) relative expression of prohormone convertase 2 (PC2) by secondary bile acids in pancreatic islets. Data are expressed as mean ± SEM. Statistical analyses were performed by one-way ANOVA followed by Tukey’s post hoc test. Multiple comparisons are summarized with lowercase letters (a > b > c). n=8-10. A, B, and C were analyzed using PICRUSt bioinformatics package software. Abbreviations: CA, cholate; CDCA, chenodeoxycholate; DCA, deoxycholate; LCA, lithocholate; UDCA, ursodeoxycholate.

Subsequently, a Phylogenetic Investigation of Communities by Reconstruction of Unobserved States (PyCrust) analysis was performed, which is designed to predict the major metabolic functions of the gut microbiota by determining the functional content of the metagenome from studies of marker genes (e.g. 16S rRNA) and whole genomes,^25^ in this study as a function of the amount of protein consumed in the diet. Interestingly, the analysis suggested that in animals fed a high protein diet, there was an increase in nitrogen metabolism (Figure 5a), particularly in the utilization of amino acids, possibly as an energy source, as observed for histidine metabolism (Figure 5b), which was also observed for other amino acids. On the other hand, the analysis also suggested a possible increase in bacterial synthesis of secondary bile acids with increasing dietary protein content (Figure 5c). To determine whether this proposed increase in secondary bile acid synthesis detected by PyCrust actually translated into an increase in their production, fecal concentrations of primary and secondary bile acids were determined. Analysis revealed that there was no difference in the concentrations of total bile acids or primary bile acids in mice fed 6%, 20%, or 50% dietary protein (Figure 5d,e), and this was associated with no change in FGF15 abundance in ileum and colon, since primary bile acids are ligands of LXR1α, a transcription factor that regulates *Fgf15* expression (Figure 5f,g). However, there was a significant increase in the concentration of the fecal secondary bile acids deoxycholic acid (DCA), lithocholic acid (LCA), and ursodeoxycholic acid (UDCA) (Figure 5h,i) with increasing dietary protein concentration. Interestingly, the serum concentration of secondary bile acids significantly increased by 4.5-fold in mice fed 50% dietary protein compared with those fed 20% dietary protein. Whereas, the concentration of primary bile acid did not differ between these groups (Figure 5j).

Secondary bile acids play various roles in the body, but there is no evidence that secondary bile acids can play a role in amino acid catabolism directly or indirectly activating AADE. On the other hand, the expression of AADE has been shown to be stimulated by circulating levels of glucagon.^11^ Previous evidence has shown that this hormone is increased in the circulation by the consumption of a high protein diet or during fasting.^9,26^ As expected, glucagon levels were significantly increased in mice fed a 50% protein diet (Figure 5k). Therefore, we investigated whether glucagon secretion was stimulated by the presence of secondary bile acids, because previous evidence showed that i.p. injection of secondary bile acids stimulated glucagon release,^27^ but some other studies with pancreatic islets did not confirm this evidence.^28^ We performed studies in isolated pancreatic islets, and in agreement with the negative results of previous studies, secondary bile acids alone were not able to increase glucagon secretion. On the other hand, there is evidence that some amino acids are glucagon secretagogues, such as arginine.^29^ Indeed, our results showed that this amino acid stimulated glucagon secretion from pancreatic islets. Surprisingly, we found that incubation of pancreatic islets with arginine in the presence of secondary bile acids synergistically increased glucagon secretion (Figure 5l), suggesting that an interaction between secretagogues amino acids and secondary bile acids is involved in promoting glucagon secretion from pancreatic islets. There is evidence that secondary bile acids are ligands for the Takeda G protein-coupled receptor 5 (TGR5), which activates the transcription of prohormone convertase enzymes that cleave proglucagon.^30^ Interestingly, our study clearly showed that the expression of the enzyme prohormone convertase 2 (PC2) increased after consumption of a high-protein diet (Figure 5m) to produce active glucagon, which partially explains the significant increase in the concentration of circulating glucagon.

### 2.6. The increase in circulating glucagon was associated with an increase in the expression of amino acid degrading enzymes

**Figure 6.**
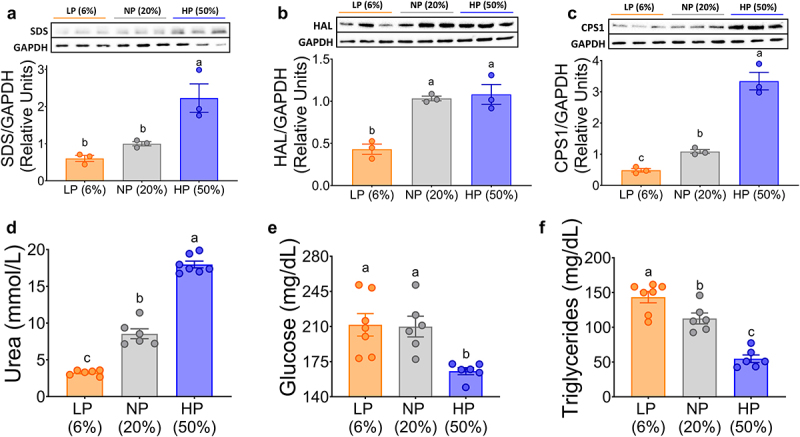
Expression of amino acid catabolizing enzymes (AACE) in mouse liver and increased postprandial urea depend on the amount of dietary protein. Densitometric analysis and blots of protein abundance of (a) SDS, (b) CPS1 and (c) HAL, GAPDH was the structural protein control. (d, e and f) Measurement of postprandial biochemical parameters. Data are presented as mean ± SEM. Statistical analyses were performed by two-way ANOVA followed by Tukey’s post hoc test (A-J). Multiple comparisons are summarized with small letters (a ≠ b ≠ c). n=5-10.

As previously shown, glucagon is an activator of AADE expression. As expected, the protein abundance of AADE, particularly serine dehydratase (SDS) and histidase (HAL), increased with increasing dietary protein concentration (Figure 6a,b). This was also evident for some urea cycle enzymes such as carbamoyl phosphate synthetase 1 (CPS1) (Figure 6c). As a consequence, we observed a decrease in the concentration of several circulating amino acids after 10 days of dietary treatment with a high-protein diet (Supplementary Fig. S1A), that was accompanied by a significant increase in serum urea concentration (Figure 6d). Interestingly, there was a decrease in serum glucose and triglyceride concentrations after consumption of a high-protein diet (Figure 6e,f).

### 2.7. Antibiotic treatment partially compensated for the differences in body weight and composition and gut microbiota after consumption of diets with different protein concentrations

**Figure 7.**
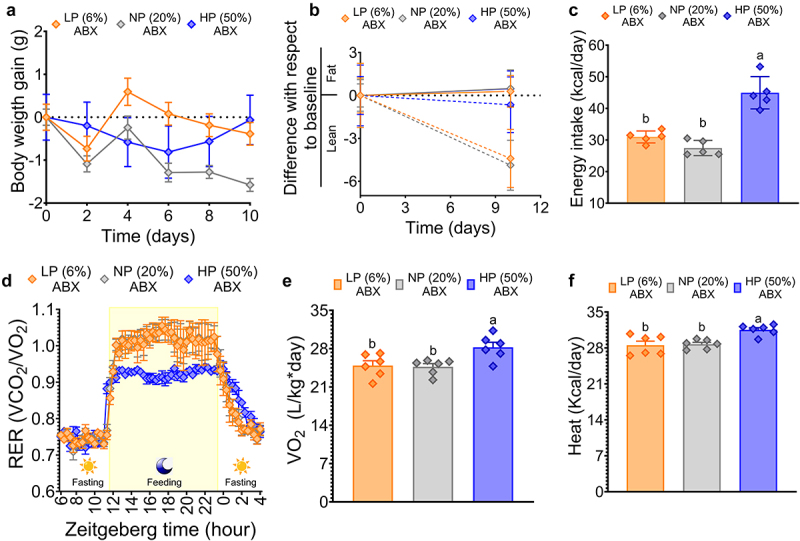
Antibiotic depletion of the gut microbiota reduced the effect of dietary protein concentration on reducing body fat mass and increasing energy expenditure. (a) Body weight gain, (b) fat mass and lean mass, (c) energy intake, (d) respiratory exchange ratio (RER), (e) oxygen consumption, and (f) heat after dietary protein consumption. LP (low protein), NP (normal protein), HP (high protein). Data are presented as mean ± SEM. Statistical analyses were performed by two-way ANOVA followed by Tukey’s post hoc test (for c and e,f). Multiple comparisons are summarized with small letters (a ≠ b ≠ c). n=5-6.

To investigate the influence of antibiotics on gut microbiota and amino acid catabolism, we conducted a parallel study in which mice were exposed to different levels of dietary protein (6%, 20% or 50%) in the presence of antibiotics. Notably, antibiotic treatment effectively abolished the differential weight gain that would typically occur with varying levels of dietary protein, resulting in almost no net weight gain over the 10-day period (Figure 7a).

However, specific differences emerged within these groups; mice consuming 6% dietary protein had a reduction in lean body mass compared to those consuming 20% and 50% dietary protein, without significant change in fat mass between groups. (Figure 7b). These changes occurred despite the fact that mice on the 50% protein diet had a higher energy intake than those on the 6% or 20% protein diets (Figure 7c). In addition, the respiratory exchange ratio (RER) in the high-protein diet group increased to 0.9 (Figure 7d), and oxygen consumption and heat production showed increases of 14% and 10%, respectively, compared to the groups fed 6% or 20% dietary protein (Figure 7e,f).

**Figure 8.**
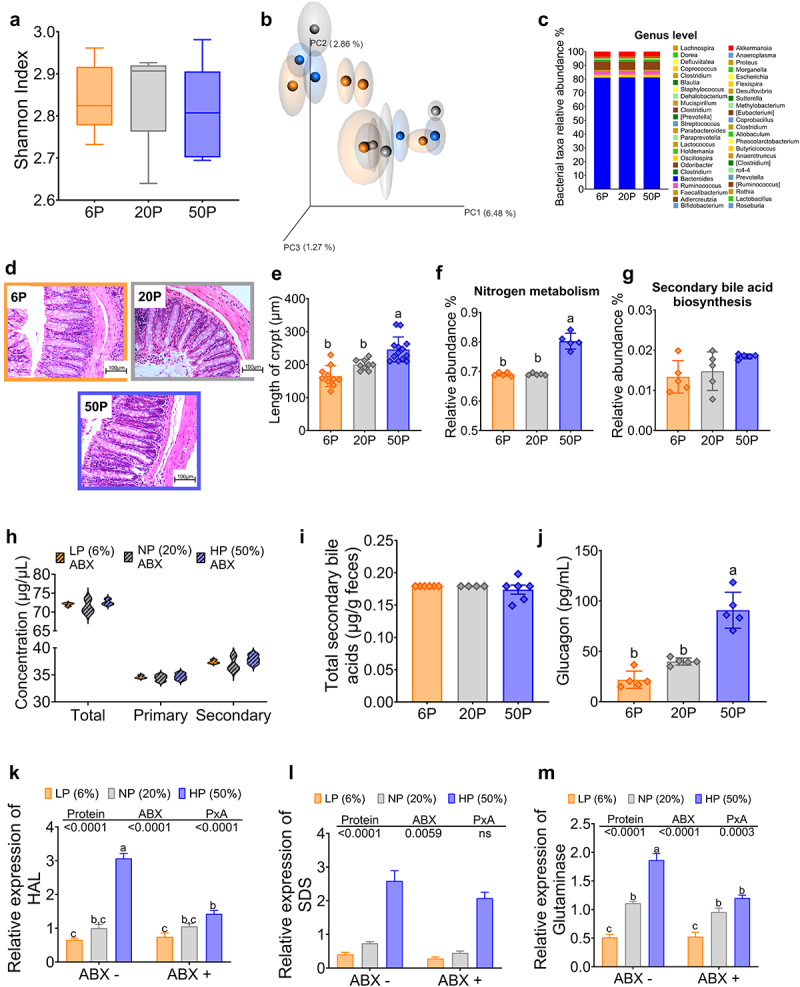
Gut microbiota depletion with antibiotics modifies the effect of dietary protein concentration on nitrogen metabolism, total secondary bile acids, and serum glucagon concentration. (a) Comparative alpha diversity in the gut microbiota, (b) principal coordinate analysis of beta diversity, (c) genus level, (d) histological sections of ascending colon collected from the indicated group of animals fed different protein concentrations and treated with antibiotic cocktail given in water consisting of antibiotic ampicillin salt/sodium (1 g mL-1) and neomycin (0.5 g mL-1) (n=10). (f) Nitrogen metabolism, (g) Relative abundance of secondary bile acid biosynthesis, (h) Serum bile acid concentration, (i) Fecal total secondary bile acids, (j) Serum glucagon concentration, Relative expression of (k) Histidase (HAL), (l) Serine dehydratase (SDS), and (m) Glutaminase. Data are presented as mean ± SEM. Statistical analysis was performed by two-way ANOVA followed by Tukey’s post hoc test (for A and E-I), n=5-10. Multiple comparisons are indicated by small letters (a ≠ b ≠ c). LP (low protein), NP (normal protein), HP (high protein). F and G were analyzed by PICRUSt.

**Figure 9.**
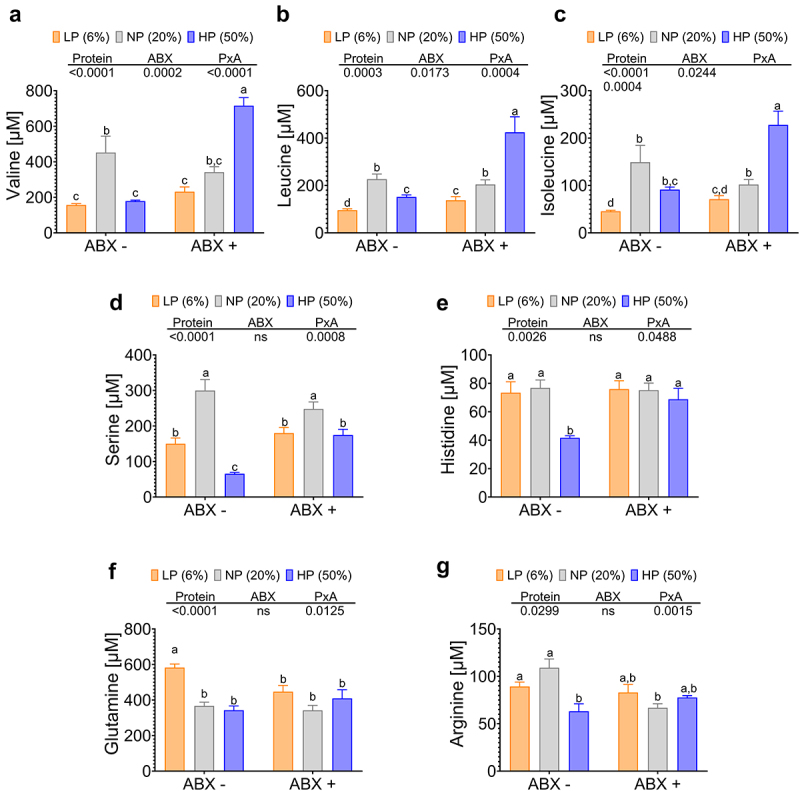
Circulating concentrations of amino acids were modified by the antibiotic treatment in mice fed different concentrations of dietary protein. (a) Valine, (b) Leucine, (c) Isoleucine, (d) Serine, (e) Histidine, (f) Glutamine, and (g) Arginine. Data are presented as mean ± SEM. Statistical analyses were performed by two-way ANOVA followed by Tukey’s post-hoc test; statistical significance was set at p < 0.05.

It is noteworthy that, with the presence of antibiotics, there was a decrease in alpha diversity and the differences between the groups fed different concentrations of dietary proteins were abolished as observed in the beta diversity of the gut microbiota (Figure 8a,b). Furthermore, the taxonomic composition at the genus level of the gut microbiota appeared quite similar in the groups fed diets containing 6%, 20% or 50% protein (Figure 8c). However, one interesting finding was that mice fed a 50% protein diet had increased crypt length in the colon (Figure 8d,e), indicating possible hyperproliferative crypts. An intriguing result of the Pycrust analysis was that the administration of antibiotics significantly decreased nitrogen metabolism with respect to mice without antibiotics, although nitrogen metabolism was higher in the group fed 50% protein with respect to the groups fed 6 or 20% dietary protein (Figure 8f), and on the other hand there was no significant difference in metabolism related to secondary bile acid biosynthesis (Figure 8g). In fact, fecal secondary bile acid concentrations were significantly reduced with antibiotic treatment, and there was no difference in serum and fecal secondary bile acid concentrations between groups (Figure 8h,i). This was accompanied by an increase in the amount of fecal total protein concentration (Supplementary Fig. S1A). As a consequence, we found that this influenced circulating levels of glucagon, as consumption of a protein-rich diet increased serum glucagon levels, but this increase was approximately 84% lower compared with those observed in mice without antibiotic treatment (Figure 8j), indicating that antibiotic administration reduced the abundance of bacteria involved in secondary bile acid production, possibly limiting glucagon secretion. In fact, qPCR analysis revealed the absence of *Parabacteroides distasonis* in fecal samples, despite the consumption of a high-protein diet. As a result, the expression of hepatic AADE, such as HAL, SDS or Glutaminase was significantly reduced in mice fed 50% protein diet in the presence of antibiotics compared to mice fed the same diet without antibiotics (Figure 8k–m). The decrease in AADE expression as a result of antibiotic treatment led to an increase in circulating amino acid levels in part due to a reduction in amino acid catabolism when mice were fed a high-protein diet compared with serum amino acid levels in mice without antibiotic treatment consuming the same diet (Figure 9a–g). Collectively, these observations highlight the complex interplay between dietary protein, gut microbiota, and metabolic outcomes and shed light on the complex relationships among these factors.

### 2.8. Consumption of a high-protein diet altered the taxonomy of the gut microbiota and increased the concentration of fecal secondary bile acids in humans

We were particularly interested in whether the effects observed in mice as a result of increased dietary protein consumption could be replicated in humans. To this end, we conducted a clinical study in healthy subjects to determine whether the changes observed in mice had analogous effects in humans in which dietary protein could alter the gut microbiota and secondary bile acids. Table 1 describes the demographic characteristics of the study subjects. The mean age of the participants was 30.8 ± 5.8 years, and 57% were female.

**Table 1.** Clinical, biochemical, and hormonal parameters of all subjects.

Variables	N=19
	Mean
Sex	
Women/Men	11/8
Age, y	30.8 ± 1.31
Weight, kg	63.9 ± 2.02
BMI, kg/m^2^	23.5 ± 0.50
Body fat mass, kg	19.1 ± 2.70
Lean body mass, kg	26.0 ± 1.28
SBP, mmHg	105 ± 2.73
DBP, mmHg	69.2 ± 1.31
Glucose, mg/dL	95.4 ± 1.78
Total cholesterol, mg/dL	167 ± 7.13
Triglycerides, mg/dL	105 ± 8.80
HDL-C, mg/dL	45.5 ± 2.81
LDL-C, mg/dL	104 ± 5.95
Urea, mmol/L	4.68 ± 0.23
Glucagon, pg/mL	6.64 ± 2.45

BMI, body mass index; SBP, systolic blood pressure; DBP, diastolic blood pressure; HDL-C, HDL cholesterol; LDL-C, LDL cholesterol.

**Figure 10.**
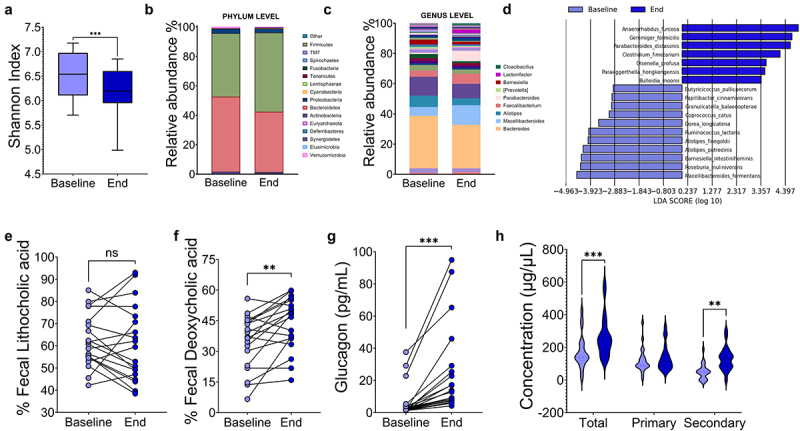
Consumption of a high-protein diet altered the taxonomy of the gut microbiota, increasing secondary bile acids and circulating glucagon in humans. (a) Comparative alpha diversity of the gut microbiota. Taxonomy of the gut microbiota at the (b) phylum level and (c) genus level. (d) Linear discriminant analysis (LDA) of the gut microbiota at the species level. Concentration of fecal secondary bile acids (e) lithocholic and (f) deoxycholic. (g) Serum glucagon concentration, (h) Serum bile acids concentration. Data are presented as mean ± SEM. Statistical analyses were performed by one-way ANOVA followed by Tukey’s post-hoc test; statistical significance was set at p < 0.05.

Interestingly, we observed that consumption of a high-protein diet reduced the alpha diversity of the gut microbiota similar to that observed in mice (Figure 10a). In addition, there were changes in the taxonomy of the gut microbiota at the phylum and genus level (Figure 10b,c). LDA analysis revealed that consumption of a high-protein diet significantly increased the abundance of several anaerobic species, including *Anaerorhabdus furcosa*, *Gemmiger formicilis*, *Clostridium fimetarium*, *Olsenella profuse*, *Paraeggerthella hongkongensis*, and *Bulleidia moorei*. Notably, we also observed an increase in the abundance of *Parabacteroides distasonis*, which was also elevated in the gut microbiota of mice fed a 50% protein diet (Figure 10d). Notably, this bacterium is associated with the synthesis of secondary bile acids. Therefore, we investigated whether the consumption of a high protein diet increases the levels of secondary bile acids.

To gain a more complete understanding of the potential effects of acute high-protein intake on secondary bile acids, we conducted a targeted metabolomic study using liquid chromatography-mass spectrometry. The aim was to detect differences in bile acid composition in stool samples collected before and after a high protein intake. The study revealed a marked increase in secondary bile acids, particularly deoxycholic acid (DCA), after high protein intake (Figure 10e,f). This increase was associated with a substantial and significant increase in glucagon levels after high protein intake, from 6.64 pg/mL to 24.7 pg/mL (Figure 10g), reflecting a similar trend to that observed in the animal model. This finding partially supports the concept that secondary bile acids may play a role in the regulation of elevated circulating glucagon levels (Figure 10h).

Finally, we compared baseline measurements with those obtained after an acute high-protein intake and observed an increase in urea levels from 4.68 mmol/L to 5.37 mmol/L (Table 2). In addition, consumption of a high-protein diet decreased visceral fat and slightly increased lean body mass, and also decreased total cholesterol and tended to decrease serum triglycerides (Table 2).

**Table 2.** Clinical, biochemical, and hormonal parameters of all subjects before and after acute protein intake.

Variables	Day 1	Day 14	*P*
	Mean	Mean	
Weight, kg	63.9 ± 2.02	63.7 ± 1.93	0.27
BMI, kg/m^2^	23.5 ± 0.50	23.5 ± 0.48	0.53
Body fat mass, kg	19.1 ± 2.70	16.4 ± 0.70	0.25
Lean body mass, kg	26.0 ± 1.28	26.3 ± 1.21	0.15
SBP, mmHg	105 ± 2.73	105 ± 2.68	0.85
DBP, mmHg	69.2 ± 1.31	70.7 ± 1.43	0.24
Glucose, mg/dL	95.4 ± 1.78	96.9 ± 2.46	0.46
Total cholesterol, mg/dL	167 ± 7.13	144 ± 7.4	0.01
Triglycerides, mg/dL	105 ± 8.80	85.0 ± 8.17	0.01
HDL-C, mg/dL	45.5 ± 2.81	46.9 ± 2.91	0.28
LDL-C, mg/dL	104 ± 5.95	97.7 ± 5.87	0.12
Urea, mmol/L	4.68 ± 0.23	5.37 ± 0.23	0.01
Glucagon, pg/mL	6.64 ± 2.45	24.7 ± 6.44	0.01

BMI, body mass index; SBP, systolic blood pressure; DBP, diastolic blood pressure; HDL-C, HDL cholesterol; LDL-C, LDL cholesterol.

## 3. Discussion

The catabolism of the excess of circulating amino acids is essential to maintain homeostasis in the organism in terms of nitrogen metabolism, allowing continuous protein synthesis according to the temporal requirements, as well as the production of several nitrogenous molecules, but at the same time preventing the potential collateral effects of the excess of amino acids.^6^ In the present study, we now recognize that the gut microbiota plays a role in the catabolism of the excess of amino acids in the liver. Previous studies have suggested that gut bacteria can use excess amino acids as a source of energy. This study and others have shown that this process can alter the population of certain gut bacteria.^14,31–33^ These changes can lead to the production of metabolites, such as secondary bile acids, which can trigger specific metabolic responses in the body.

Interestingly, we observed that this adaptation of the gut microbiota to the consumption of a high-protein diet was accompanied by several phenotypic changes observed in the mouse model, including a reduction in body weight gain, but preventing the decrease in lean body mass, attenuating the increase in body fat, compared to mice fed an adequate amount of dietary protein, effects observed in previous clinical studies.^34,35^ These results are consistent with previous studies in animal models or in humans that a high protein diet prevents obesity and preserves lean body mass.^22,36^ This response to increased dietary protein was in part associated with increased energy expenditure, as previously described.^37^ Moreover, increased dietary protein reduces RER to 0.7 indicating a switch in oxidation from carbohydrates to fat. This can be explained by the different proportion of carbohydrate and fat in these diets but also by the increase in specific amino acids. Specifically, the high protein content provides a continuous supply of amino acids that can replenish the Krebs cycle intermediates, contributing to an increase in fatty acid oxidation. For instance, leucine treatment of C2C12 myotube significantly increases fatty acid oxidation.^38^ Furthermore, the increase in anaplerotic reactions fed by aspartate and BCAA oxidation enhanced endurance and resistance to muscle fatigue with greater lipid oxidation in mice deficient of the histone deacetylase 3 (HDAC3).^39^

Our study also showed that an additional adaptation occurred in the intestine, particularly in the colon segment, since our observation after an RNAseq analysis, showed a differential transcriptomic gene expression profile between mice fed a high-protein diet and those fed adequate or low protein diet. The results showed a high increase in colonic *Atg7* expression involved in the autophagy process,^40^ *Slc16A3* a transporter of short chain fatty acids, *HNF4-α* involved in intestinal cell differentiation,^41^ *Smad3* associated with the transcription of *TGFα* regulating the proliferation of the intestinal epithelial barrier,^42^ and *Ncoa2* involved in cell proliferation and reduced apoptosis.^43^ As a consequence, the intestinal epithelium of mice fed a high protein diet showed large epithelial crypts as a result in the expression of genes involved in cell proliferation and improvement of the epithelial barrier, a result consistent with previous studies in rats and humans.^44–46^ Interestingly, there was an increase in the expression of *HIF1α* in the colon when mice were fed a high protein diet. It has been described that increased expression of *HIF1α* in the intestine is associated with a local anaerobic condition.^47,48^ Our results clearly showed that consumption of a high protein diet resulted in a significant increase in colonic anaerobiosis. There is evidence that consumption of a high-protein diet can increase the synthesis of short-chain fatty acids by the gut microbiota, which can be used as an energy substrate by the intestine, but also intestinal cells can use an excess of some amino acids as an energy source.^19,47,49^ Indeed, in the present study, a significant increase in mitochondrial oxidative capacity was observed in isolated colon mitochondria. This suggests that the elevated oxidative capacity in the colon is due to a significant increase in the utilization of metabolic substrates, extensively using oxygen and generating an anaerobic state.^48,50^

There is clear evidence that the anaerobic state in the colon can promote a change in the taxonomy of the gut microbiota.^51^ This effect was observed in our study, where we found that the gut microbiota after consumption of a high protein diet differentially modified the relative abundance of gut bacteria compared to consumption of an adequate or low protein diet, as observed by beta diversity analysis. There was a significant increase in some species of the gut microbiota with a high protein diet, including *Escherichia coli*, *Clostridium cocleatum*, *Mucispirillum schaedleri* and *Parabacteroides distasonis*. Interestingly, *Parabacteroides distasonis* has been observed to thrive on higher protein intakes due to its ability to utilize protein as a nutrient source. After passing through the esophagus and gastric lumen, dietary proteins are hydrolyzed in the small intestine by proteases and peptidases. Although the process is quite efficient, some nitrogenous products not absorbed in the small intestine cross the ileocecal junction and can be found in the large intestine. The undigested peptides and amino acids are usually not absorbed by colonocytes, but are utilized by the gut microbiota to form intermediate metabolites and other end products such as p-cresol from tyrosine, phenylpropionate, phenylacetate from phenylalanine, and indole skatole from tryptophan among others, and this will depend on the amount of dietary protein consumed.^6^ *Parabacteroides distasonis* has the enzymatic machinery to break down and metabolize the excess of amino acids. Therefore, as protein intake increases, it provides more substrate for these bacteria to grow and multiply in the intestinal environment. The enzymes possessed by *Parabacteroides distasonis* include: proteases, aminotransferases, and decarboxylases. These enzymes cleave peptide bonds within proteins, breaking them down into smaller peptides and amino acids that can be further metabolized, facilitate the transfer of amino groups between amino acids and keto acids, a process crucial for amino acid metabolism and synthesis, and in particular, *Parabacteroides distasonis* possess decarboxylases that remove carboxyl groups from amino acids, producing biogenic amines or other metabolites.^52,53^ Together, these enzymes allow *Parabacteroides distasonis* to utilize amino acids as a nutrient source when protein intake is increased, contributing to its growth and survival in the intestinal environment as observed in the present study. This relationship is evidence of the complex interactions between diet and the composition of the gut microbiota and highlights how dietary changes can influence microbial populations in the gut ecosystem. Interestingly, in this study, when humans were fed a high-protein diet, there was an increase in the abundance of several anaerobic bacteria, including *Parabacteroides distasonis*.

Some studies have shown that *Parabacteroides distasonis* is involved in the synthesis of succinate, but also in the synthesis of secondary bile acids.^54^ Therefore, we investigated whether the consumption of a high protein diet increased the levels of secondary bile acids.

Primary bile acids are synthesized in the liver from cholesterol and conjugated with glycine or taurine to form bile salts, which are stored in the gallbladder. During digestion, these bile salts are released into the small intestine to help emulsify and absorb fats. In the large intestine, intestinal bacteria convert primary bile acids to secondary bile acids, such as cholic acid to deoxycholic acid and chenodeoxycholic acid to lithocholic acid. Some secondary bile acids are reabsorbed and returned to the liver for reuse in the bile cycle. As observed in the present study, we found for the first time that consumption of a high protein diet increases the formation of secondary bile acids. Secondary bile acids are involved in several functions, including fat digestion, cholesterol and energy metabolism, and have also been implicated in cell signaling via the farnesoid X receptor (FXR) or Takeda G protein-coupled receptor 5 (TGR5).^28,55–58^ For example, secondary bile acids can increase the expression of uncoupling protein 1 (UCP1) through TGR5 during browning of white adipose tissue.^28,59^ TGR5 is expressed in several organs and tissues, including the pancreas.^60^ In fact, mice overexpressing *TGR5* have an increased insulin secretion.^61^ Nevertheless, its role on glucagon has been less studied. For instance, alpha cells of pancreatic islets also express *TGR5*, and it has been known that its activation can induce the expression of prohormone convertase enzymes (PC).^30^ Proglucagon is produced by the cell-specific expression of PC1 and PC2 to produce GLP-1 and glucagon, respectively.^62,63^ There is evidence that a bile acid analog can increase GLP-1 in alpha cells by increasing the expression of PC1 via TGR5.^30^ Interestingly, our study in mice demonstrated that the increase in secondary bile acids can induce the expression of PC2 and increase circulating levels of glucagon. This effect was associated with an increase in circulating amino acids that are secretagogues of glucagon, such as arginine, as we observed in our studies with pancreatic islets.^29^ Thus, there is a synergistic effect between the increase in secondary bile acids from the gut microbiota and the increase in certain amino acids to promote glucagon secretion. This effect was observed in humans fed a high protein diet, as we found an increase in the formation of secondary bile acids by the gut microbiota, particularly deoxycholic acid, which was accompanied by an increase in circulating levels of glucagon.

Interestingly, in our animal study, the use of antibiotics partially reduced the effect of a high protein diet on the circulating levels of glucagon, possibly due to the abolition of secondary bile acid synthesis. These results suggest that some amino acids after consumption of a high-protein diet increased the expression of several AADE and urea cycle enzymes to prevent the excessive elevation of amino acids. Interestingly, the use of antibiotics in the animal model partially prevented the increased expression of these enzymes, indicating the role of the gut microbiota in the regulation of amino acid catabolism through a decrease in secondary bile acid synthesis and its consequence on the modulation of glucagon secretion.

In conclusion, our study reveals a complex interaction between gut microbiota and hepatic amino acid catabolism, highlighting a communication pathway facilitated by secondary bile acids. We found that a protein-rich diet increases the abundance of *Parabacteroides distasonis*, a bacterium responsible for the production of secondary bile acids. These bile acids in the presence of amino acids induce the release of glucagon from the pancreas, which activates gene expression of amino acid-degrading enzymes to eliminate the excess circulating amino acids, serving as a protective regulatory response to the potential toxicity of a high-protein diet. Future research, including studies in germ-free mice and human trials with *Parabacteroides distasonis* as a probiotic or the use of a secondary bile acid supplement, will further validate our findings on amino acid metabolism. Our study highlights the dynamic relationship between gut microbiota, amino acid metabolism and hormone regulation, and provides valuable insights into physiological responses to dietary protein intake.

## 4. Materials and methods

### 4.1. Animal model and dietary treatments

Male C57BL/6J mice 7-9 weeks old were bred and used in the 4th generation, maintained on a 12 h light/12 h dark cycle at 21°C and fed ad libitum for 10 days on one of three diets containing different concentrations of dietary proteins: a low-protein (6%), an adequate protein (20%), or a high-protein (50%) diet according to the AIN (AIN-93) dietary recommendations for laboratory rodents^64^ (Supplementary Table S1). Three mice per cage were housed

To evaluate the effect of gut microbiota on amino acid catabolism, each group fed a different protein concentration was divided with and without antibiotics. A total of 120 mice were used and divided into 20 animals for each dietary treatment with or without antibiotic. Three mice per cage were maintained to minimize the caged effect on the gut microbiota study. Antibiotic treatment was started on day 1. The antibiotic cocktail administered in water consisted of ampicillin/sodium salt (1 g/L) and neomycin (0.5 g/L) [25]. At the end of the study, during the feeding period, after a 12-h fast and a 3-h refeeding period, a blood sample was collected from the portal vein, and immediately centrifuged at 3000 rpm per 10 min to obtain the serum samples. Additionally, liver and intestinal samples were also collected. The samples were stored at -70°C until analysis. To examine the degree of colonic hypoxia, another group of mice fed different concentrations of dietary protein were injected intraperitoneally with pimonidazole (60 mg/kg body weight) 90 min before euthanasia.

### 4.2. Energy expenditure

Energy expenditure of C57BL/6J mice was assessed by indirect calorimetry. Animals were individually housed for 48 hours in Plexiglas cages with an open flow system connected to an Oxymax Laboratory Animal Monitoring System (CLAMS, Columbus Instruments, Columbus, OH, USA). Animals were acclimated for 24 hours, fasted for 12 hours during the light period, and fed during the dark period. Oxygen consumption (VO_2_, mL/kg/h) and CO_2_ production (VCO_2_, mL/kg/h) concentrations were monitored to calculate oxygen consumption and respiratory exchange ratio. Measurements were taken in each chamber at 22-minute intervals. Energy expenditure (EE) was calculated using the following equation: EE= (3.815 + 1.232*RER)*VO_2_.^65^

### 4.3. Body composition analysis

The body composition of each mouse was evaluated at the end of the study using magnetic resonance imaging (EchoMRI, Echo Medical Systems, Houston, TX, U.S.A.) to measure lean and fat mass. Mice were placed in a thin-walled plastic cylinder with a cylindrical plastic insert to restrict movement in a quantitative magnetic resonance imaging system. Inside the cylinder, the animals were briefly exposed to a low intensity electromagnetic field (0.05 Tesla) for 2 minutes.^66^

### 4.4. Biochemical and hormonal analyses

Serum total urea and glucose were measured in a COBAS c111 photometric clinical chemistry analyzer. Serum glucagon was measured by ELISA (EHGCG, Invitrogen, Carlsbad, CA).

### 4.5. Serum amino acid analysis

The amino acid profile was determined by using high performance liquid chromatography (HPLC). An aliquot of serum was thawed and 100 μL of serum was added to 25 μL of 10% sulfosalicylic acid to deproteinize the sample. Samples were incubated for 30 min under refrigeration and centrifuged at 14,000 rpm for 15 min at 4 °C. Then, 99 μL of the supernatant was taken and 1 μL of the internal standard (norvaline; 15 mm) was added; the sample was derivatized and injected. The procedure was performed using a sampling device (Agilent; G1367F) coupled to an HPLC (Agilent 1260 Infinity, California, USA,) and a fluorescence detector (Agilent; G1321B). A ZORBAX Eclipse AAA column was used and maintained at 40 °C. Chromatographic conditions were maintained according to the technical instructions for the column.

### 4.6. Histologic analysis

Colon tissues were fixed in 10% formalin overnight, dehydrated in alcohol, cleared in xylene, and embedded in paraffin. Tissues were sectioned (4 μm) and stained with hematoxylin and eosin. Crypt length was determined using analysis software by quantifying the length of five crypts from four zones of four animals per study group.

### 4.7. Hypoxia assay

Hypoxia was measured in longitudinal sections of the colon, previously fixed in paraffin, from mice previously injected with pimonidazole, using Hypoxyprobe™ as an antibody as previously described by^67,68^ in a dilution (1:25).

### 4.8. Analysis of mitochondrial function

Mitochondrial respiration was determined in isolated mitochondria from ileum and colon using the Seahorse XFe96 Extracellular Flux Analyzer (Agilent Technologies). Mice were euthanized and 40 mg of ileum and colon were placed in cold mitochondrial isolation buffer (MIB1, 210 mM d-Manitol, 70 mm) and centrifuged at 800 x g for 10 min at 4°C. The tissue was then homogenized and centrifuged at 800 x g for 10 min at 4°C. The collected supernatant was centrifuged at 8000 x g (10 min at 4°C) and the pellet containing mitochondria was washed three times with MIB1 and resuspended in mitochondrial assay solution (MAS1, 220 mM d-mannitol, 70 mm sucrose, 10 mm KH2PO4, 5 mm MgCl2, 2 mm HEPES, 1 mm EGTA, 0. 2% BSA, pH 7.2) with the addition of substrates (10 mm glutamate and 5 mm malate). Six µg of isolated mitochondria were diluted in MAS1 buffer with substrates and loaded per well in the XFe96 plate. Platted mitochondria were centrifuged at 2000 xg for 20 min at 4°C. Oxygen consumption rate (OCR) was measured in seven technical replicates for each mouse using the following compounds: ADP (4 mm), oligomycin (2.5 µM), FCCP (3 µM), and antimycin A (1 µM)/rotenone (1 µM). Four mitochondrial respiratory states were calculated.

### 4.9. RNAseq analysis

Total RNA was extracted from intestinal sections using the guanidinium thiocyanate/cesium chloride gradient method. RNA integrity was assessed by capillary electrophoresis QIAxcel (QIAGEN, MA Germany). The corresponding 48 RNA libraries were constructed according to Illumina’s TruSeq Strand-Specific RNA sequencing library protocol (https://support.illumina.com/downloads/truseq_stranded_total_rna_sample_preparation_guide_15031048.html). The RNA libraries were sequenced on a HiSeq 2500 system at Lifesequencing S.L. Paired-end reads of 101 base pairs (within a range of 36,240,231-77,906,369 total reads) were generated on this platform. Reads obtained from Illumina were analyzed for quality control (QC), and filtering of raw data was performed using FASTQC software, and removal of contamination and adapters was performed using internal Perl scripts. Filtered reads were aligned using the Bowtie 1.1.234 aligner. Quantification and normalization for replicates were performed using eXpress 1.535 software. Total effective counts for each sample were pooled and a matrix was generated using the abundance_estimates_to_matrix.pl Perl script included in the Trinity pipeline. The resulting matrix was used as input for differential expression analysis, with the following selected parameters: p-adj/FDR = 0.05; logFC = 2; CPM = 1; differential expression analysis was performed using limma-Voom version 3.38.3, using the log2 counts per million normalization method, and DESeq2 version 1.22.2.^69^

### 4.10. Quantification of fecal secondary bile acids

Fecal samples were processed by homogenization in methanol, heating and centrifugation followed by filtration. Targeted metabolomics analysis was performed using a modified method based on that developed by Ulaszewska et al .^70^ Briefly, 2 uL of fecal sample extracts were injected into a Zorbax Eclipse C18 hPLC column (Agilent Technologies). The solvents used as mobile phases for chromatographic separation were water (A) and acetonitrile (B) (JT Baker), both containing 0.1% formic acid (Sigma Aldrich). The gradient used was as follows: 0-1.5 min 25%B, 1.5-14min 75%, 14-16.8 min 80%B, 16.8-25 min 100%, 25-27.1 min 75%B, and 27.1-28 min 25%B. Multiple Reaction Monitoring (MRM) experiments were performed in a QTRAP 6500+ (ABSciex) in negative ionization mode at -4500 V, 550°C TEM, CAD low, CUR gas 35 psi, gas 1 and gas 2 60 psi, and EP -10 V. The monitored transitions were as follows (parent ion/fragment): cholic acid mz = 407.3/407.3, ursodeoxycholic acid mz = 391.3/391.3, chenodeoxycholic acid mz = 391.3/391.3, deoxycholic acid mz = 391.3/391.3 and 345, and lithocholic acid mz = 375.3/375.3. Calibration curves were constructed using chemical standards (Sigma Aldrich) for the different metabolites diluted in methanol in the range of 0.0045-10 µM for fecal samples. The acquired data were processed using SciexOS. The concentration of all compounds was estimated linearly from the peak areas.

### 4.11. Quantification of serum secondary bile acids

The isolation of bile acids from serum was through modifications to the technique described by Batta AK.^71^ 100 µl of serum were added to hyodeoxycholic acid (100 µg in 10 µl of methanol), 100 mm acetate buffer pH 5.6 (200 µl), 1.86% EDTA (100 µl). 0.87% β-mercaptoethanol (100 µl) and 100 mg cholylglycine hydrolase and 100 mg β-glucuronidase. The resulting suspension was incubated at 37°C for 18 hours. The reaction mixture was then filtered through prewashed reverse-phase Sep-Pak C18 cartridges and the release of bile acids was eluted with 700 µl of acetone. After evaporation of the acetone, 100 µl of n-butanol and 20 µl of a 40 % solution of hydrochloric acid in dioxane were added and incubated at 60° C for 4 hours and then kept at room temperature overnight. After evaporation of the solvents at 60°C, the esterified bile acids were subjected to trimethylsilylation (TMS).

With this aim, the esterified bile acids were added 100 µl of Sil-Prep [hexamethyldisilazone-trimethylchlorosilane-pyridine (3:1:9)] and incubated for 30 min at 55°C. The solvents were evaporated at 55°C under N_2_ stream and the TMS formed were recovered in 100 µl of hexane and 5 µl were injected into the chromatographic column. The retention times and concentration of the different bile acids were calculated in relation to hyodeoxycholic acid.

An Agilent Technologies model 6890 gas chromatograph equipped with a flame ionization detection system and an injector with a split/splitless device for capillary columns was used for all separations. The chromatographic column consisted of a cyclodextrin capillary column directly bound to dimethylpolysiloxane, DB-225MS (30m X 0.25 mm I.D., df 0.25 µm) (Agilent Technologies) and hydrogen was used as carrier gas. The GC operating conditions were: injector and detector temperatures 260°C and 290°C, respectively. After injection, the oven temperature was maintained at 100°C for 2 min, and then programmed at a ramp rate of 35°C/min to reach a temperature of 278°C.

### 4.12. Total fecal protein concentration

Stool samples were dissolved in water suitable for molecular biology and homogenized using a TissueLyser at a frequency of 30 hz for 2 minutes, centrifuged at 10000 g/10 min/4 °C. The supernatant was recovered and stool protein concentration was determined according to the Lowry assay (Bio-Rad) and normalized per microgram of stool.

### 4.13. Gut microbiota sequencing 16S rRNA sequencing

From total DNA of each subject, the V3 and V4 regions of the 16S rRNA ribosomal gene were amplified using specific forward (5ʹ TCGTCGGCAGCGTCAGATGTGTA TAAGAGACAGCCTACGGGNGGCWGCAG 3ʹ) and reverse primers (5ʹ GTCTCGTGGGCTCGGAGATGTGTATAAGAGACAGGACTACHVGGTATCTAATC

C 3ʹ) containing the Illumina adapter overhang nucleotide sequences. Ampure XP beads were used to purify 16S V3-V4 amplicons, which were quantified by high-resolution capillary electrophoresis (QIAxcel, QIAGEN, Germany). The amplicon size was approximately 550 bp. An index PCR was then performed to attach the dual indices using a Nextera XT v2 kit. The amplicon was approximately 610 bp, and the concentration of double-stranded DNA was measured using a Qubit 3.0 fluorometer with a high sensitivity kit. The final library of amplicons was pooled at equimolar concentrations. Sequencing was performed on the Illumina MiSeq platform (MiSeq Reagent Kit V.3, 600 cycles) at 15 pM with 20% Phyx infection according to the manufacturer’s instructions to generate paired end reads of 300 bases in each direction.

### 4.14. Bioinformatic analysis of gut microbiota

Custom C# and Python scripts in the QIIME pipeline 1.9 software^72^ were used to analyze the taxonomic composition of the sequencing files. Sequences were subjected to quality filtering and chimera detection using Gold.fa. Operational taxonomic units (OTUs) were generated by clustering sequences with percent sequence similarity using the Usearch method in QIIME.^73^ The GreenGenes v.13.9 database was used for OTU selection, and 97% of OTUs were selected. Species richness (observed, Chao1) and alpha diversity (Shannon) were calculated, and principal coordinate analysis (PCoA) was performed using weighted and unweighted UniFrac distances. ANOSIM determined statistically significant clusters based on microbiota structural distances. Finally, PICRUSt (Phylogenetic Investigation of Communities by Reconstruction of Unobserved States) analysis revealed possible metabolic pathways activated in the gut microbiota by diets with different protein content.^25^

### 4.15. Quantitative real-time PCR (RT-qPCR)

Total RNA was extracted from pancreas with TRIzol reagent according to the manufacturer’s instructions (Thermo Fisher Scientific, California, USA), and 3 μg of total RNA was used to synthesize cDNA by reverse transcription. The cDNA samples were then subjected to quantitative real-time polymerase using SYBR Green I Master and specific primers to assess the expression of prohormone convertase 2 (PC2) using the SYBR LightCycler® 480, Roche Thermocycler. Relative mRNA expression levels were normalized to those of the internal control *36B4* gene. The sequences of the primers were *PC2* forward 5‘AGACAATGGGAAGACGGTTG3’ and reverse 5‘CTTGAAGCATAGCCGTCACA 3’; 36B4 forward 5‘AGATTCGGGATATGCTGTTGG 3’ and reverse AAAGCCTGGAAGAAGGAGGTC 3’.

In order to quantify the abundance *Parabacteroides distasonis*, fecal DNA was extracted using the QIAGEN Power Fecal DNA extraction kit according to the manufacturer’s instructions. The primers sequence was F: 5’-TGATCCCTTGTGCTGCT-3’ and R: 5’-ATCCCCCTCATTCGGA-3’ were used for PCR-based amplification. Real-time PCR was performed using SYBER Green assay (Roche). The PCR conditions included 95 °C for 3 min, then, followed by 45 cycles by amplification of denaturation at 95 °C for 10 s, annealing at 56 °C for 10 s, and extension at 72 °C for 10 s. Final extension was per 2 min.

### 4.16. Glucagon secretion

Islets were isolated from male C57BL/6J mice (8 weeks old) as previously described.^74^ Briefly, the pancreas was infused through the bile duct with Hank’s balanced salt solution (HBSS, Corning®, cat. no. 21-021-CV) with BSA 0.5 % and CaCl_2_ 0.36 mm and Collagenase type V (1.5 mg/mL; Sigma, cat. no. C9263). Then, the pancreatic tissue was removed and digested with 5 mL of supplemented HBSS for 15 min at 37 °C slightly shaken by hand every 5 min. The digested tissue was washed twice with HBSS with BSA 0.5 % and CaCl_2_ 0.36 mm by centrifugation at 900 rpm for 30 s at 4 °C and the pellet was resuspended in 15 mL of HBSS. The islets were separated by pouring the resuspended pellet in a pre-wetted 70 μm cell strainer (Corning, cat. no. 431751) and washed twice the with 25 ml of the same solution. Afterward, the cell strainer was turned upside down and the isolated islets were collected with 3 ml of RPMI 1640 medium supplemented with 10% fetal calf serum, 1 mm sodium pyruvate, 50 mm 2-mercaptoethanol, 2 mm glutamine, 10 mm HEPES, and 100 U/mL penicillin, and 100 mg/mL streptomycin. After 24 hours, the islets were handpicked under a microscope to exclude any contaminating tissue.

To evaluate glucagon secretion, 10 islets per condition were washed and incubated in Krebs balanced salt solution (NaCl 130 mm, KCl 3.4 mm, NaH_2_PO4 0.5 mm, MgSO4 1.0 mm, HEPES 10 mm, Na_2_CO_3_ 5 mm, CaCl_2_ 1.5 mm) with glucose 12 mm for 1 h at 37°C. Islets were then incubated with either 12 mm glucose (HG), 1 mm glucose (LG), 1 mm glucose + 10 mm arginine (LG+Arg), 1 mm glucose + lithocholic acid (LCA) 5 µM (LG+LCA), or glucose 1 mm + arginine 10 mm + LCA 5 µM (LG+Arg+LCA) for 2 hours at 37°C. To stop glucagon secretion, islets were placed on ice and then centrifuged at 2000 rpm for 2 min at 4°C. The medium was collected and frozen for determination of glucagon concentration using an ELISA kit (ALPCO, No. Cat. 48-GLUHU-E01). Total glucagon content was determined in an aliquot obtained by addition of acidified ethanol (75% ethanol/1.5% HCl) to the islet pellet. Glucagon secretion was expressed as the percentage of glucagon secreted into the media with respect to the sum of total and secreted glucagon content. Experiments were performed in quadruplicate and repeated to ensure reproducibility.

### 4.17. Western blot analysis

Total protein was extracted from the liver using lysis buffer containing a protease inhibitor cocktail (Roche, Germany). Twenty micrograms of extracted protein were loaded onto SDS-PAGE gels (10%) and transferred to a polyvinylidene difluoride membrane (Bio-Rad, Hercules, CA). Blocking with 5% nonfat dry milk and antibody incubation were performed. The membranes were further probed with antibodies against HNF4α (sc-8987 1:1000), SDS, HAL (sc-133646 1:1000), CPS1 (sc-376190 1:5000), GAPDH (sc-365062 1:1000) (Santa Cruz Biotechnology, Dallas, TX, and Waltham, MA, USA). Proteins of interest were detected with goat anti-mouse IgG H&L antibody (ab6789 1:15000) or rabbit anti-goat IgG antibody (sc-2768, 1:3500) from Santa Cruz Biotechnology and Abcam, USA. Antibody detection reactions were performed using Immobilon Western chemiluminescent substrate from HRP (Millipore, Temecula, CA). Chemiluminescence was digitized using a ChemiDoc MP imaging system (Bio-Rad, Hercules, CA) and analyzed using ImageJ software.

### 4.18. Human studies and dietary protein intervention

A before-and-after clinical trial was conducted to evaluate the effect of a high-protein diet. Participants were recruited between August and September 2023. Participants who met all eligibility criteria were selected and enrolled. Inclusion criteria were as follows: age over 18 years, with a body mass index (BMI) below 30 kg/m^2^. Exclusion criteria were subjects diagnosed with any chronic disease, taking any medication or antibiotics 3 months prior to the study, subjects with creatinine >1.3 mg/dL for men and >1 mg/dL for women, and urea nitrogen >20 mg/dL, and smokers. The study consisted of a 14-day intervention divided into two phases. The first phase of 1 week (0 - 7 days) was a diet with total energy intake based on their total energy expenditure measured by indirect calorimetry, which included a macronutrient distribution of 50% carbohydrate, 30% fat and 20% protein. And the second phase of 1 week (7 - 14 days) recommended a diet with total energy intake based on their total energy expenditure measured by indirect calorimetry, and a macronutrient distribution of 40% carbohydrate, 30% fat and 20% protein and added 10% more protein supplemented with calcium caseinate. Each participant underwent a health assessment. Anthropometric measurements were taken, including height and weight (Seca 700, Germany). Body mass index (BMI) was calculated by dividing weight (kg) by height squared (m^2^). On days 0 and 14, anthropometric and biochemical parameters were assessed. Fasting blood was collected in the morning from a peripheral vein and centrifuged at 3,000 rpm for 10 minutes. The resulting serum was aliquoted and stored at -80°C until analysis. Serum biochemical and hormonal parameters were measured is indicated above. Stool samples were collected for subsequent determination of secondary bile acids and gut microbiota at baseline and final visits. Stool samples were collected in a 5 ml cryovial prefilled with 95% ethanol and stored at -80 °C until analysis.

## 5. Statistical analysis

Animal results obtained in this study are presented as mean ± SEM. One-way ANOVA followed by Tukey’s post hoc test for multiple comparisons and variable interactions was used to examine the effects of treatments. A two-way ANOVA was used to assess the interaction between the protein intake and antibiotic use for AADE and circulating levels of amino acids followed by Tukey’s post hoc test. For anthropometric, biochemical, and hormonal parameters in humans, the paired Student’s t-test analysis was used to compare between baseline and final visit. Statistical analyses were performed using SPSS version 22.0 software (SPSS Inc., Chicago, IL, U.S.A.) or GraphPad Prism 9, and statistical significance was set at p < 0.05.

## Supplementary Material

Supplemental Material

## Data Availability

The 16S rRNA gene and RNAseq sequencing raw sequence reads (fastq) are available at the NCBI Sequence Read Archive with BioProject IDs PRJNA1064983, PRJNA1063863, PRJNA1065444. The available data are accessible to the reviewers at the following links: https://dataview.ncbi.nlm.nih.gov/object/PRJNA1065444?reviewer=6ou2emvuofvhg0vut5g00vc4s, https://dataview.ncbi.nlm.nih.gov/object/PRJNA1064983?reviewer=qfi265kms1tlf7ibqus2fjjhl6, and https://dataview.ncbi.nlm.nih.gov/object/PRJNA1063863?reviewer=9b8m04ed5pp31k7d9ouqu6da31. Other data supporting the results of this study are available from the corresponding author upon reasonable request.

## References

[1] Venn BJ. Macronutrients and Human Health for the 21st Century. Nutrients. 2020;12(8):12. doi:10.3390/nu12082363. PMC7468865 32784664

[2] Wu G. Dietary protein intake and human health. Food Funct. 2016;7(3):1251–26. doi:10.1039/C5FO01530H. 26797090

[3] Blachier F, Beaumont M, Portune KJ, Steuer N, Lan A, Audebert M, Khodorova N, Andriamihaja M, Airinei G, Benamouzig R, et al. High-protein diets for weight management: interactions with the intestinal microbiota and consequences for gut health. A position paper by the my new gut study group. Clin Nutr. 2019;38(3):1012–1022. doi:10.1016/j.clnu.2018.09.016. 30274898

[4] Krieger JW, Sitren HS, Daniels MJ, Langkamp-Henken B. Effects of variation in protein and carbohydrate intake on body mass and composition during energy restriction: a meta-regression 1. Am J Clin Nutr. 2006;83(2):260–274. doi:10.1093/ajcn/83.2.260. 16469983

[5] Spring S, Premathilake H, DeSilva U, Shili C, Carter S, Pezeshki A. Low protein-high carbohydrate diets alter energy balance, gut microbiota composition and blood metabolomics profile in young pigs. Sci Rep. 2020;10(1):3318. doi:10.1038/s41598-020-60150-y. 32094453 PMC7040010

[6] Torres N, Tobon-Cornejo S, Velazquez-Villegas LA, Noriega LG, Aleman-Escondrillas G, Tovar AR. Amino acid catabolism: an overlooked area of metabolism. Nutrients. 2023;15(15):15. doi:10.3390/nu15153378. PMC10421169 37571315

[7] Fouillet H, Juillet B, Bos C, Mariotti F, Gaudichon C, Benamouzig R, Tome D. Urea-nitrogen production and salvage are modulated by protein intake in fed humans: results of an oral stable-isotope-tracer protocol and compartmental modeling. Am J Clin Nutr. 2008;87(6):1702–1714. doi:10.1093/ajcn/87.6.1702. 18541559

[8] Rose AJ. Role of peptide hormones in the adaptation to altered dietary protein intake. Nutrients. 2019;11(9):11. doi:10.3390/nu11091990. PMC6770041 31443582

[9] Tovar AR, Ascencio C, Torres N. Soy protein, casein, and zein regulate histidase gene expression by modulating serum glucagon. Am J Physiol Endocrinol Metab. 2002;283(5):E1016–E1022. doi:10.1152/ajpendo.00398.2001. 12376330

[10] MacDonald PE, Rorsman P. Metabolic messengers: glucagon. Nat Metab. 2023;5(2):186–192. doi:10.1038/s42255-022-00725-3. 36639733 PMC12494092

[11] Aleman G, Ortiz V, Langley E, Tovar AR, Torres N. Regulation by glucagon of the rat histidase gene promoter in cultured rat hepatocytes and human hepatoblastoma cells. Am J Physiol Endocrinol Metab. 2005;289(1):E172–E179. doi:10.1152/ajpendo.00584.2004. 15741241

[12] Tobon-Cornejo S, Vargas-Castillo A, Leyva-Martinez A, Ortiz V, Noriega LG, Velazquez-Villegas LA, Aleman G, Furosawa-Carballeda J, Torres N, Tovar AR. PPARα/RXRα downregulates amino acid catabolism in the liver via interaction with HNF4α promoting its proteasomal degradation. Metabolism. 2021;116:154705. doi:10.1016/j.metabol.2021.154705. 33422545

[13] Velazquez-Villegas LA, Charabati T, Contreras AV, Aleman G, Torres N, Tovar AR. PPARα downregulates hepatic glutaminase expression in mice fed diets with different protein:carbohydrate ratios. J Nutr. 2016;146(9):1634–1640. doi:10.3945/jn.116.232868. 27466601

[14] Wu S, Bhat ZF, Gounder RS, Mohamed Ahmed IA, Al-Juhaimi FY, Ding Y, Bekhit AEA. Effect of dietary protein and processing on gut microbiota—a systematic review. Nutrients. 2022;14(3):14. doi:10.3390/nu14030453. PMC8840478 35276812

[15] Snelson M, Clarke RE, Nguyen TV, Penfold SA, Forbes JM, Tan SM, Coughlan MT. Long term high protein diet feeding alters the microbiome and increases intestinal permeability, systemic inflammation and kidney injury in mice. Mol Nutr Food Res. 2021;65(8):e2000851. doi:10.1002/mnfr.202000851. 33547877

[16] Wu L, An R, Lan T, Tang Z, Xu Y, Peng X, Pang J, Sun W, Shi B, Tang Q, et al. Isocaloric diets with varying protein levels affected energy metabolism in young adult Sprague-Dawley rats via modifying the gut microbes: A lipid imbalance was brought on by a diet with a particularly high protein content. J Nutr Biochem. 124:109534. doi:10.1016/j.jnutbio.2023.109534. 37977404

[17] Rowland I, Gibson G, Heinken A, Scott K, Swann J, Thiele I, Tuohy K. Gut microbiota functions: metabolism of nutrients and other food components. Eur J Nutr. 2018;57(1):1–24. doi:10.1007/s00394-017-1445-8. PMC5847071 28393285

[18] Bartlett A, Kleiner M. Dietary protein and the intestinal microbiota: an understudied relationship. iScience. 2022;25(11):105313. doi:10.1016/j.isci.2022.105313. 36339270 PMC9626677

[19] Davila AM, Blachier F, Gotteland M, Andriamihaja M, Benetti PH, Sanz Y, Tome D. Intestinal luminal nitrogen metabolism: role of the gut microbiota and consequences for the host. Pharmacol Res. 2013;68(1):95–107. doi:10.1016/j.phrs.2012.11.005. 23183532

[20] Russell WR, Duncan SH, Scobbie L, Duncan G, Cantlay L, Calder AG, Anderson SE, Flint HJ. Major phenylpropanoid-derived metabolites in the human gut can arise from microbial fermentation of protein. Mol Nutr Food Res. 2013;57(3):523–535. doi:10.1002/mnfr.201200594. 23349065

[21] Holecek M. Side effects of amino acid supplements. Physiol Res. 2022;71:29–45. doi:10.33549/physiolres.934790. 35043647 PMC8997670

[22] Moon J, Koh G. Clinical evidence and mechanisms of high-protein diet-induced weight loss. J Obes Metab Syndr. 2020;29(3):166–173. doi:10.7570/jomes20028. 32699189 PMC7539343

[23] Munro HM. Nutritional consequences of excess amino acid intake. Adv Exp Med Biol. 1978;105:119–129. 103372 10.1007/978-1-4684-3366-1_8

[24] Wu G. Intestinal mucosal amino acid catabolism. J Nutr. 1998;128(8):1249–1252. doi:10.1093/jn/128.8.1249. 9687539

[25] Langille MG, Zaneveld J, Caporaso JG, McDonald D, Knights D, Reyes JA, Clemente JC, Burkepile DE, Vega Thurber RL, Knight R, et al. Predictive functional profiling of microbial communities using 16S rRNA marker gene sequences. Nat Biotechnol. 2013;31(9):814–821. doi:10.1038/nbt.2676. 23975157 PMC3819121

[26] Stern JH, Smith GI, Chen S, Unger RH, Klein S, Scherer PE. Obesity dysregulates fasting-induced changes in glucagon secretion. J Endocrinol. 2019;243(2):149–160. doi:10.1530/JOE-19-0201. 31454790 PMC6994388

[27] Kuhre RE, Wewer Albrechtsen NJ, Larsen O, Jepsen SL, Balk-Moller E, Andersen DB, Deacon CF, Schoonjans K, Reimann F, Gribble FM, et al. Bile acids are important direct and indirect regulators of the secretion of appetite- and metabolism-regulating hormones from the gut and pancreas. Mol Metab. 2018;11:84–95. doi:10.1016/j.molmet.2018.03.007. 29656109 PMC6001409

[28] Perino A, Demagny H, Velazquez-Villegas L, Schoonjans K. Molecular physiology of bile acid signaling in health, disease, and aging. Physiol Rev. 2021;101(2):683–731. doi:10.1152/physrev.00049.2019. 32790577

[29] Maruszczak K, Rasmussen C, Ceutz FR, Orgaard A, Elmelund E, Richter MM, Holst JJ, Winther-Sørensen M, Wewer Albrechtsen NJ. Arginine-induced glucagon secretion and glucagon-induced enhancement of amino acid catabolism are not influenced by ambient glucose levels in mice. Am J Physiol Endocrinol Metab. 2022;323(3):E207–E214. doi:10.1152/ajpendo.00122.2022. 35830690

[30] Kumar DP, Asgharpour A, Mirshahi F, Park SH, Liu S, Imai Y, Nadler JL, Grider JR, Murthy KS, Sanyal AJ. Activation of transmembrane bile acid receptor TGR5 modulates pancreatic islet α cells to promote glucose homeostasis. J Biol Chem. 2016;291(13):6626–6640. doi:10.1074/jbc.M115.699504. 26757816 PMC4807250

[31] Gao J, Liu Z, Wang C, Ma L, Chen Y, Li T, Deng W. Effects of dietary protein level on the microbial composition and metabolomic profile in postweaning piglets. Oxid Med Cell Longev. 2022;2022:1–13. doi:10.1155/2022/3355687. PMC8986435 35401925

[32] Liu X, Blouin JM, Santacruz A, Lan A, Andriamihaja M, Wilkanowicz S, Benetti P-H, Tomé D, Sanz Y, Blachier F, et al. High-protein diet modifies colonic microbiota and luminal environment but not colonocyte metabolism in the rat model: the increased luminal bulk connection. Am J Physiol Gastrointest Liver Physiol. 2014;307(4):G459–G470. doi:10.1152/ajpgi.00400.2013. 24970777

[33] Mu C, Yang Y, Luo Z, Zhu W. Temporal microbiota changes of high-protein diet intake in a rat model. Anaerobe. 2017;47:218–225. doi:10.1016/j.anaerobe.2017.06.003. 28629947

[34] Westerterp-Plantenga MS, Lemmens SG, Westerterp KR. Dietary protein – its role in satiety, energetics, weight loss and health. Br J Nutr. 2012;108(Suppl 2):S105–S112. doi:10.1017/S0007114512002589. 23107521

[35] Westerterp-Plantenga MS, Nieuwenhuizen A, Tome D, Soenen S, Westerterp KR. Dietary protein, weight loss, and weight maintenance. Annu Rev Nutr. 2009;29(1):21–41. doi:10.1146/annurev-nutr-080508-141056. 19400750

[36] Chalvon-Demersay T, Blachier F, Tome D, Blais A. Animal models for the study of the relationships between diet and obesity: a focus on dietary protein and estrogen deficiency. Front Nutr. 2017;4:5. doi:10.3389/fnut.2017.00005. 28373974 PMC5357654

[37] Bray GA, Redman LM, de Jonge L, Covington J, Rood J, Brock C, Mancuso S, Martin CK, Smith SR. Effect of protein overfeeding on energy expenditure measured in a metabolic chamber. Am J Clin Nutr. 2015;101(3):496–505. doi:10.3945/ajcn.114.091769. 25733634

[38] Liang C, Curry BJ, Brown PL, Zemel MB. Leucine modulates mitochondrial biogenesis and SIRT1-AMPK signaling in C2C12 myotubes. J Nutr Metab. 2014;2014:1–11. doi:10.1155/2014/239750. PMC4220583 25400942

[39] Emmett MJ, Lim HW, Jager J, Richter HJ, Adlanmerini M, Peed LC, Briggs ER, Steger DJ, Ma T, Sims CA, et al. Histone deacetylase 3 prepares brown adipose tissue for acute thermogenic challenge. Nature. 2017;546(7659):544–548. doi:10.1038/nature22819. 28614293 PMC5826652

[40] Collier JJ, Suomi F, Olahova M, McWilliams TG, Taylor RW. Emerging roles of ATG7 in human health and disease. EMBO Mol Med. 2021;13(12):e14824. doi:10.15252/emmm.202114824. 34725936 PMC8649875

[41] Ullah MS, Davies AJ, Halestrap AP. The Plasma membrane lactate transporter MCT4, but Not MCT1, Is up-regulated by hypoxia through a HIF-1α-dependent mechanism. J Biol Chem. 2006;281(14):9030–9037. doi:10.1074/jbc.M511397200. 16452478

[42] Planchon SM, Martins CA, Guerrant RL, Roche JK. Regulation of intestinal epithelial barrier function by TGF-beta 1. Evidence for its role in abrogating the effect of a T cell cytokine. J Immunol. 1994;153(12):5730–5739. doi:10.4049/jimmunol.153.12.5730. 7989770

[43] Triki M, Lapierre M, Cavailles V, Mokdad-Gargouri R. Expression and role of nuclear receptor coregulators in colorectal cancer. World J Gastroenterol. 2017;23(25):4480–4490. doi:10.3748/wjg.v23.i25.4480. 28740336 PMC5504363

[44] Beaumont M, Andriamihaja M, Armand L, Grauso M, Jaffrezic F, Laloe D, Moroldo M, Davila A-M, Tomé D, Blachier F, et al. Epithelial response to a high-protein diet in rat colon. BMC Genomics. 2017;18(1):116. doi:10.1186/s12864-017-3514-z. 28137254 PMC5282643

[45] Beaumont M, Portune KJ, Steuer N, Lan A, Cerrudo V, Audebert M, Dumont F, Mancano G, Khodorova N, Andriamihaja M, et al. Quantity and source of dietary protein influence metabolite production by gut microbiota and rectal mucosa gene expression: a randomized, parallel, double-blind trial in overweight humans. Am J Clin Nutr. 2017;106(4):1005–1019. doi:10.3945/ajcn.117.158816. 28903954

[46] Reese AT, Pereira FC, Schintlmeister A, Berry D, Wagner M, Hale LP, Wu A, Jiang S, Durand HK, Zhou X, et al. Microbial nitrogen limitation in the mammalian large intestine. Nat Microbiol. 2018;3(12):1441–1450. doi:10.1038/s41564-018-0267-7. 30374168 PMC6264799

[47] Kelly CJ, Zheng L, Campbell EL, Saeedi B, Scholz CC, Bayless AJ, Wilson K, Glover L, Kominsky D, Magnuson A, et al. Crosstalk between microbiota-derived short-chain fatty acids and intestinal epithelial HIF augments tissue barrier function. Cell Host Microbe. 2015;17(5):662–671. doi:10.1016/j.chom.2015.03.005. 25865369 PMC4433427

[48] Zheng L, Kelly CJ, Colgan SP. Physiologic hypoxia and oxygen homeostasis in the healthy intestine. A review in the theme: cellular responses to hypoxia. Am J Physiol Cell Physiol. 2015;309(6):C350–60. doi:10.1152/ajpcell.00191.2015. 26179603 PMC4572369

[49] Zhou C, Li L, Li T, Sun L, Yin J, Guan H, Wang L, Zhu H, Xu P, Fan X, et al. SCFAs induce autophagy in intestinal epithelial cells and relieve colitis by stabilizing HIF-1α. J Mol Med (Berl). 2020;98(8):1189–1202. doi:10.1007/s00109-020-01947-2. 32696223

[50] Shelton CD, Byndloss MX, Richardson AR. Gut epithelial metabolism as a key driver of intestinal dysbiosis associated with noncommunicable diseases. Infect Immun. 2020;88(7):88. doi:10.1128/IAI.00939-19. PMC7309626 32122941

[51] Litvak Y, Byndloss MX, Baumler AJ. Colonocyte metabolism shapes the gut microbiota. Science. 2018;362(6418). doi:10.1126/science.aat9076. PMC6296223 30498100

[52] Albenberg LG, Wu GD. Diet and the intestinal microbiome: associations, functions, and implications for health and disease. Gastroenterology. 2014;146(6):1564–1572. doi:10.1053/j.gastro.2014.01.058. 24503132 PMC4216184

[53] Wu GD, Chen J, Hoffmann C, Bittinger K, Chen YY, Keilbaugh SA, Bewtra M, Knights D, Walters WA, Knight R, et al. Linking long-term dietary patterns with gut microbial enterotypes. Science. 2011;334(6052):105–108. doi:10.1126/science.1208344. 21885731 PMC3368382

[54] Wang K, Liao M, Zhou N, Bao L, Ma K, Zheng Z, Wang Y, Liu C, Wang W, Wang J, et al. Parabacteroides distasonis alleviates obesity and metabolic dysfunctions via production of succinate and secondary bile Acids. Cell Rep. 2019;26(1):222–35 e5. doi:10.1016/j.celrep.2018.12.028. 30605678

[55] Chiang JYL, Ferrell JM. Bile acid receptors FXR and TGR5 signaling in fatty liver diseases and therapy. Am J Physiol Gastrointest Liver Physiol. 2020;318(3):G554–G573. doi:10.1152/ajpgi.00223.2019. 31984784 PMC7099488

[56] Fiorucci S, Distrutti E. Bile acid-activated receptors, intestinal microbiota, and the treatment of metabolic disorders. Trends Mol Med. 2015;21(11):702–714. doi:10.1016/j.molmed.2015.09.001. 26481828

[57] Pathak P, Liu H, Boehme S, Xie C, Krausz KW, Gonzalez F, Chiang JYL. Farnesoid X receptor induces Takeda G-protein receptor 5 cross-talk to regulate bile acid synthesis and hepatic metabolism. J Biol Chem. 2017;292(26):11055–11069. doi:10.1074/jbc.M117.784322. 28478385 PMC5491788

[58] Sayin SI, Wahlstrom A, Felin J, Jantti S, Marschall HU, Bamberg K, Angelin B, Hyötyläinen T, Orešič M, Bäckhed F. Gut microbiota regulates bile acid metabolism by reducing the levels of tauro-beta-muricholic acid, a naturally occurring FXR antagonist. Cell Metab. 2013;17(2):225–235. doi:10.1016/j.cmet.2013.01.003. 23395169

[59] Velazquez-Villegas LA, Perino A, Lemos V, Zietak M, Nomura M, Pols TWH, Schoonjans K. TGR5 signalling promotes mitochondrial fission and beige remodelling of white adipose tissue. Nat Commun. 2018;9(1):245. doi:10.1038/s41467-017-02068-0. 29339725 PMC5770450

[60] Sorrentino G, Perino A, Yildiz E, El Alam G, Bou Sleiman M, Gioiello A, Pellicciari R, Schoonjans K. Bile acids signal via TGR5 to activate intestinal stem cells and epithelial regeneration. Gastroenterology. 2020;159(3):956–68 e8. doi:10.1053/j.gastro.2020.05.067. 32485177

[61] Thomas C, Gioiello A, Noriega L, Strehle A, Oury J, Rizzo G, Macchiarulo A, Yamamoto H, Mataki C, Pruzanski M, et al. TGR5-mediated bile acid sensing controls glucose homeostasis. Cell Metab. 2009;10(3):167–177. doi:10.1016/j.cmet.2009.08.001. 19723493 PMC2739652

[62] Rouille Y, Westermark G, Martin SK, Steiner DF. Proglucagon is processed to glucagon by prohormone convertase PC2 in alpha TC1-6 cells. Proc Natl Acad Sci USA. 1994;91(8):3242–3246. doi:10.1073/pnas.91.8.3242. 8159732 PMC43552

[63] Whalley NM, Pritchard LE, Smith DM, White A. Processing of proglucagon to GLP-1 in pancreatic α-cells: is this a paracrine mechanism enabling GLP-1 to act on β-cells? J Endocrinol. 2011;211(1):99–106. doi:10.1530/JOE-11-0094. 21795304

[64] Reeves PG, Nielsen FH, Fahey GC Jr. AIN-93 purified diets for laboratory rodents: final report of the American Institute of Nutrition ad hoc writing committee on the reformulation of the AIN-76A rodent diet. J Nutr. 1993;123(11):1939–1951. doi:10.1093/jn/123.11.1939. 8229312

[65] Nie Y, Gavin TP, Kuang S. Measurement of resting energy metabolism in mice using oxymax open circuit indirect calorimeter. Bio Protoc. 2015;5(18). doi:10.21769/BioProtoc.1602. PMC4751037 26878029

[66] Nixon JP, Zhang M, Wang C, Kuskowski MA, Novak CM, Levine JA, Billington CJ, Kotz CM. Evaluation of a quantitative magnetic resonance imaging system for whole body composition analysis in rodents. Obesity (Silver Spring). 2010;18(8):1652–1659. doi:10.1038/oby.2009.471. 20057373 PMC2919581

[67] Cousins FL, Murray AA, Scanlon JP, Saunders PT. Hypoxyprobe™ reveals dynamic spatial and temporal changes in hypoxia in a mouse model of endometrial breakdown and repair. BMC Res Notes. 2016;9(1):30. doi:10.1186/s13104-016-1842-8. 26780953 PMC4717617

[68] Liu S, Hur YH, Cai X, Cong Q, Yang Y, Xu C, Bilate AM, Gonzales KAU, Parigi SM, Cowley CJ, et al. A tissue injury sensing and repair pathway distinct from host pathogen defense. Cell. 2023;186(10):2127–43 e22. doi:10.1016/j.cell.2023.03.031. 37098344 PMC10321318

[69] Maya-Maldonado K, Cime-Castillo J, Maya-Lucas O, Argotte-Ramos R, Rodriguez MC, Lanz-Mendoza H. Transcriptome analysis uncover differential regulation in cell cycle, immunity, and metabolism in Anopheles albimanus during immune priming with Plasmodium berghei. Dev Comp Immunol. 2021;120:104046. doi:10.1016/j.dci.2021.104046. 33600838

[70] Ulaszewska MM, Mancini A, Garcia-Aloy M, Del Bubba M, Tuohy KM, Vrhovsek U. Isotopic dilution method for bile acid profiling reveals new sulfate glycine-conjugated dihydroxy bile acids and glucuronide bile acids in serum. J Pharm Biomed Anal. 2019;173:1–17. doi:10.1016/j.jpba.2019.05.002. 31100508

[71] Batta AK, Salen G, Rapole KR, Batta M, Earnest D, Alberts D. Capillary gas chromatographic analysis of serum bile acids as the n-butyl ester–trimethylsilyl ether derivatives. J Chromatogr B Biomed Sci Appl. 1998;706(2):337–341. doi:10.1016/S0378-4347(97)00564-1. 9551821

[72] Caporaso JG, Kuczynski J, Stombaugh J, Bittinger K, Bushman FD, Costello EK, Fierer N, Peña AG, Goodrich JK, Gordon JI, et al. QIIME allows analysis of high-throughput community sequencing data. Nat Methods. 2010;7(5):335–336. doi:10.1038/nmeth.f.303. 20383131 PMC3156573

[73] Edgar RC. Search and clustering orders of magnitude faster than BLAST. Bioinformatics. 2010;26(19):2460–2461. doi:10.1093/bioinformatics/btq461. 20709691

[74] Li DS, Yuan YH, Tu HJ, Liang QL, Dai LJ. A protocol for islet isolation from mouse pancreas. Nat Protoc. 2009;4(11):1649–1652. doi:10.1038/nprot.2009.150. 19876025

