# Supplementary material for: Increased dietary protein stimulates amino acid catabolism via the gut microbiota and secondary bile acid production
Source: Gut Microbes. 2025 Feb 20;17(1):2465896. doi: 10.1080/19490976.2025.2465896 (PMC11849929; doi:10.1080/19490976.2025.2465896)
Supplement: Supplemental Material [file KGMI_A_2465896_SM4274.zip › Supplementary_Table_1_diets clean.docx]

| Supplementary Table 1. Experimental diet composition | | | |
| --- | --- | --- | --- |
| Ingredients (g/100g diet) | **LP** | **NP** | **HP** |
| Cornstarch | 48.6 | 39.7 | 20.8 |
| Casein | 6 | 20 | 50 |
| Dextrinized cornstarch | 16.14 | 13.26 | 6.95 |
| Sucrose | 12.22 | 10 | 5.2 |
| Soybean oil | 7 | 7 | 7 |
| Fiber Celluose | 5 | 5 | 5 |
| Mineral Mix AIN-93G-MX | 3.5 | 3.5 | 3.5 |
| Vitamin Mix AIN-93-VX | 1 | 1 | 1 |
| L-Cystine | 0.3 | 0.3 | 0.3 |
| Choline bitartrate | 0.25 | 0.25 | 0.25 |
| Tert-butylhydroquinone | 0.0014 | 0.0014 | 0.0014 |
| Carbohydrates calories | 307.84 | 251.84 | 131.8 |
| Fat Calories | 63 | 63 | 63 |
| Protein Calories | 24 | 80 | 200 |
| Total Calories | 394.8 | 394.8 | 394.8 |
